# A genome wide transcriptional model of the complex response to pre-TCR signalling during thymocyte differentiation

**DOI:** 10.18632/oncotarget.5796

**Published:** 2015-09-22

**Authors:** Hemant Sahni, Susan Ross, Alessandro Barbarulo, Anisha Solanki, Ching-In Lau, Anna Furmanski, José Ignacio Saldaña, Masahiro Ono, Mike Hubank, Martino Barenco, Tessa Cromp­ton

**Affiliations:** ^1^ Institute of Child Health, University College London, London WC1N 1EH, UK

**Keywords:** pre-TCR, thymus, foetal thymic organ cultures, DP, genome wide transcriptional modelling, Immunology Section, Immune response, Immunity

## Abstract

Developing thymocytes require pre-TCR signalling to differentiate from CD4−CD8− double negative to CD4+CD8+ double positive cell. Here we followed the transcriptional response to pre-TCR signalling in a synchronised population of differentiating double negative thymocytes. This time series analysis revealed a complex transcriptional response, in which thousands of genes were up and down-regulated before changes in cell surface phenotype were detected. Genome-wide measurement of RNA degradation of individual genes showed great heterogeneity in the rate of degradation between different genes. We therefore used time course expression and degradation data and a genome wide transcriptional modelling (GWTM) strategy to model the transcriptional response of genes up-regulated on pre-TCR signal transduction. This analysis revealed five major temporally distinct transcriptional activities that up regulate transcription through time, whereas down-regulation of expression occurred in three waves. Our model thus placed known regulators in a temporal perspective, and in addition identified novel candidate regulators of thymocyte differentiation.

## INTRODUCTION

In this study we investigate the whole genome changes in gene transcription in response to a developmental stimulus through time, in a synchronized population of differentiating cells, in their physiological environment. Many studies have used analysis of the steady-state transcriptome to investigate the changes in gene expression that take place during development and differentiation. In general such work has been limited to taking ‘snap-shots’ of changes in transcription at a particular time point in development, or has examined gene expression in populations of cells developing along a particular cell-lineage by comparing expression in sequential populations defined by the cell-surface expression of developmentally regulated markers. In this study, we followed the transcriptomes of developing thymocytes in response to pre-TCR signal transduction, under conditions where differentiation is synchronous. To capture the changes in transcription in the physiological microenvironment of the thymus, we used foetal thymic organ cultures (FTOCs) and measured the heterogeneity in gene expression and degradation using microarrays.

T lymphocytes develop in the thymus, which provides an essential environment for T cell fate specification, and for the differentiation of multipotent progenitor cells into functional T cells. This cellular developmental programme has been well defined in terms of stepwise changes in cell surface markers and rearrangement of TCR gene loci, as the developing thymocytes move through different thymic microenvironments. Briefly, the most immature progenitors lack expression of both the CD4 or CD8 co-receptors, and are termed DN (Double Negative) thymocytes. The DN cells differentiate to become CD4+CD8+ double positive (DP) cells, which give rise to single positive (SP) CD4 or CD8 αβ T cells [1, 2]. The DN population can be subdivided into four major subsets by the ordered expression of CD44 and CD25: CD44+CD25− (DN1); CD44+CD25+ (DN2); CD44−CD25+ (DN3); CD44−CD25− (DN4) [3]. Notch signalling is essential for this developmental programme. Notch proteins promote T cell fate in BM precursors entering the thymus, and in mice deficient in Notch1 T-cell lineage specification/commitment fails in the thymus, while forced expression of Notch1 leads to generation of T cells in the bone marrow [2, 4, 5]. Notch signals maintain survival of early DN cells until the DN3 stage, but following pre-TCR signalling, they are abruptly down-regulated as Id3 protein rapidly rises and supresses E2A-mediated Notch [6].

The TCR-β gene locus is in the germ line configuration until the DN2 stage, when its rearrangement begins, requiring the Recombinase activating genes (Rag)1 and Rag2. The expression of a rearranged TCR-β chain together with pre-Tα and CD3 molecules to form a pre-TCR complex represents a critical check point in T cell development [7]. Signals from the pre-TCR complex are essential to initiate the developmental transition, and thus to cease further recombination of the TCR-β loci, for differentiation from DN3 to subsequent stages of development, for expansion, and to rescue developing T cells from apoptosis. Despite identification of several molecules that regulate this process [2, 6, 8–12], an understanding of the temporal regulation of the genome-wide molecular events and the molecular regulators underpinning this transition remains restricted, and new experimental approaches that allow the identification of potential novel developmental regulators are required.

Here, we used time series analysis of the synthesis and degradation data with a genome-wide transcriptional modelling strategy, which has previously been validated in cell lines [13]. This approach dissected the transcriptional response to the pre-TCR signal into five principal activities, whereas down-regulation of gene expression occurred in three main waves. The analysis temporally integrated all molecular mediators downstream of the pre-TCR, including those already known by genetic approaches.

T acute lymphoblastic leukaemia (T-ALL) arises from the malignant transformation of thymocytes at early stages of their development, and frequently involves dysregulated Notch1 signalling and dysregulation of the developmental processes associated with pre-TCR signalling [14–20]. Our study is thus important to our understanding of T-ALL, in addition to the information it provides about the molecular regulators of physiological T cell development, and it places genes previously identified to be functionally important in T-ALL in the temporal context of pre-TCR signal transduction.

## RESULTS

### Pre-TCR signal transduction in Rag1−/− FTOC

**Figure 1:**
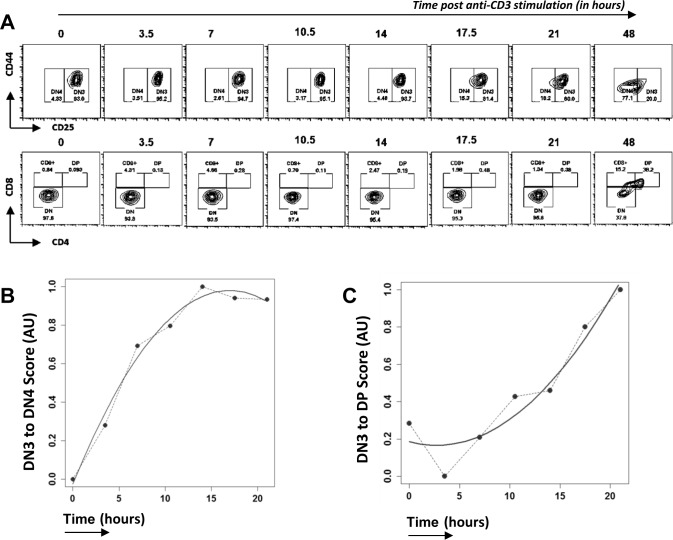
Time course analysis of transcriptional changes in Rag1−/− FTOC on anti-CD3 treatment **A.** Differentiation of thymocytes from E17.5 Rag1−/− embryos in FTOCs after anti-CD3ε stimulation analysed by flow cytometry through time, at time points stated. Contour plots show staining for CD44 and CD25 on cells gated to be CD4^−^CD8^−^(upper panel) and staining for CD4 and CD8 (lower panel). Th1.2+ cells were purified from these cultures at the time points shown, RNA extracted, and gene expression measured by microarray. **B.** and **C.** Cannonical Correspondence Analysis (CCA) of the transcriptome of anti-CD3 treated Rag1−/− thymocytes through time (in hours): **B.** plotted against a scale of DN3 to DN4, generated from transcriptome data from sorted DN3 and DN4 thymocytes from the Immgen database; **C.** plotted on a scale of DN3 to DP generated from transcriptome data from sorted DN3 and DP thymocytes from the Immgen database. Dotted lines join the values at each time point, and solid lines show the best fitting curve.

The pre-TCR complex can oligomerize and signal in the absence of a ligand [21], but its signal transduction is dependent on the CD3-ε and CD3-γ chains [22]. Rag-deficient thymocytes arrested at DN3 can be induced to differentiate to DP cell by anti-CD3 treatment, and this has been used extensively experimentally to investigate pre-TCR function and study the transition of developing cells in a synchronous manner [23]. We treated E17.5 Rag1−/− FTOCs with anti-CD3 antibody and followed their differentiation for 2 days (Figure 1A). RNA was isolated every 3.5 hours for 21 hours from sorted Thy1.2+ cells and microarrays performed to obtain whole genome transcriptomic data. At 21 hours post treatment ∼18% of cells were DN4, and none had progressed to the DP stage, while ∼38% of cells were DP after 2 days. Although such cultures have been widely used to study this developmental transition, we first validated our experimental system, and tested if the changes in transcription measured by microarray through time, mirrored those observed in phenotypically defined differentiated thymocyte populations (DN3, DN4, DP). To do this we used Canonical Correspondence Analysis (CCA) [24–26] to compare our microarray data to external publically available microarray data sets from sorted DN3, DN4 and DP thymocyte populations (Immgen database [27]). CCA is a mathematical methodology that has been developed by ecologists and sociologists for multivariate analysis, and it allows comparison of microarray data from a given experiment to externally generated microarray data sets. We generated a scale of DN3→DN4 (Figure 1B) and DN3→DP (Figure 1C) using the Immgen microarray data, and used this as a gradient for CCA of our own microarray data sets through time. The transcriptome of the anti-CD3-treated thymocytes increased in similarity to the defined DN4 and DP transcriptomes through time. During the first 10 hours after treatment, the transcriptional signature of the thymocytes changed rapidly from that of DN3 cells towards that of DN4 cells, and plateaued in the transcriptional signature of ‘DN4-ness’ at around 12 hours (Figure 1B). In contrast, on the scale of DN3 to DP, the gene expression scored closer to DN3 for the first 10 hours, and then increased steeply in the transcriptional signature of ‘DP-ness’ until the end of the experiment (Figure 1C). This analysis thus demonstrated that anti-CD3 stimulation of Rag−/− thymocytes reproduces the physiological transcriptional response to pre-TCR signalling, as measured in sorted populations of DN4 and DP thymocytes. Interestingly, the CCA showed that the changes in transcription induced by the stimulus occurred rapidly and were detectable long before the cell surface phenotypes of DN4 or DP populations were apparent.

### Pre-TCR signal transduction initiates a complex molecular process

**Figure 2:**
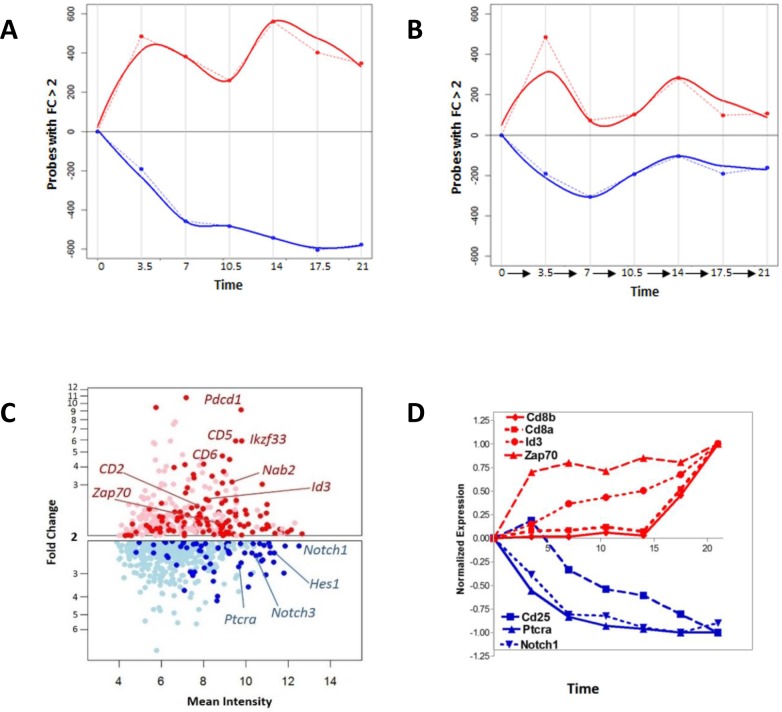
The pre-TCR initiates a complex molecular response **A.** Number of genes with fold change >2 with respect to time zero, plotted through the time course of the experiment, showing up- and down-regulated genes. The number of up-regulated genes are shown in red, and are shown graphically in the positive half of the x-axis, whereas the number of down-regulated genes are shown in blue, and are illustrated graphically in the negative half of the x-axis. Dotted lines join the values at each time point, and solid lines show the best fitting curve. **B.** Number of genes with fold change >2 at transitions between successive measurements. The number of up-regulated genes between successive measurements are shown in red, and are shown graphically in the positive half of the x-axis, whereas the number of down-regulated genes between successive measurements are shown in blue, and are illustrated graphically in the negative half of the x-axis. Dotted lines join the values at each time point, and solid lines show the best fitting curve. **C.** Genes with fold change >2 with respect to time zero at seven hours after anti-CD3 stimulation. Selected genes have been marked on the plot. Up-regulated genes are shown in red, and down-regulated genes are shown in blue. Light red and light blue dots represent those not annotated to any gene on the current Affymetrix 1.0 ST definitions. **D.** The expression pattern of selected transcripts through time in the experiment. Up-regulated genes are shown in red, and down-regulated genes are shown in blue.

In order to understand the magnitude of changes downstream of the pre-TCR signal, we measured the number of transcripts induced or repressed following the anti-CD3 stimulus (Figure 2A–2B). Compared to control (t=0), 925 transcripts had a fold change of greater than two at the end of the time course. Assessment of the differentially regulated transcript number over time revealed a complex picture in which the up-regulated genes follow a biphasic time course, and an early response is followed by a second flux in the later time points (Figure 2A–2B). Within the first 7 hours following stimulation, there was a two-fold up-regulation of *Id3*, known to interact with the E2A transcription factor and decrease its occupancy from its target enhancer elements (Figure 2C–2D) [28, 29]. Other transcripts up-regulated at 7 hours included those for the immune-modulatory cell surface protein CD5, the transcription factor Nab2 and the tyrosine kinase Zap-70, while transcript levels for CD25 protein were found to be down-regulated; all previously reported to be downstream of pre-TCR signal transduction [30]. Analysis of *Cd4* and *Cd8* expression, the defining markers of the DP population, revealed up-regulation of *Cd8β* and *Cd8α* from 17 hours onwards, while *Cd4* expression remained very low (not shown) throughout the time-course. The expression pattern of *CD8α* and *Cd8β* is distinct from other known up-regulated genes such as *Zap70* or *Id3*, which were up-regulated earlier (Figure 2D).

### Expression analysis of down-regulated genes

**Figure 3:**
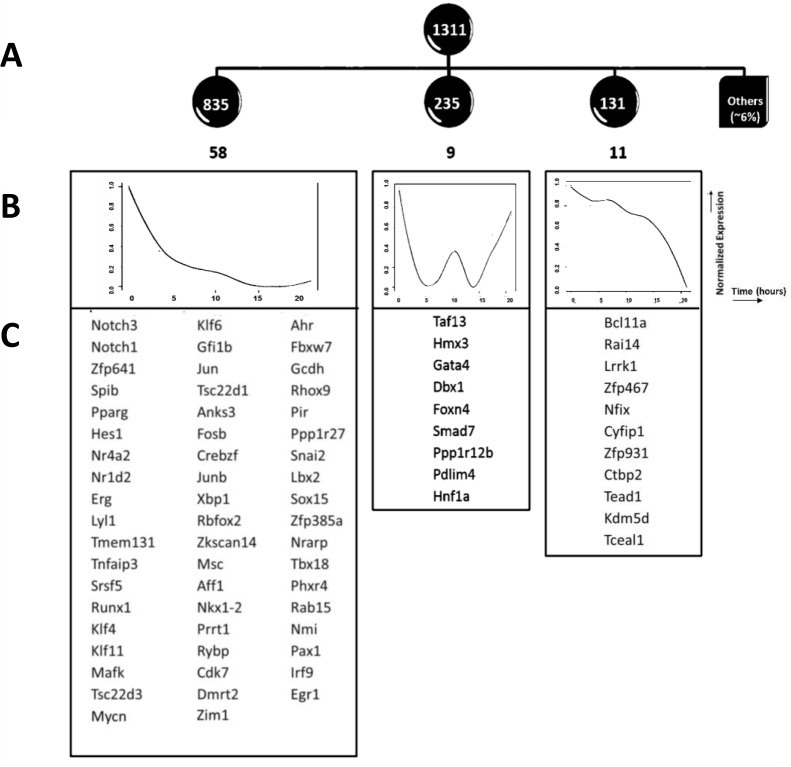
Down regulation of expression of genes following pre-TCR signal transduction **A.** Expression based analysis of down-regulated genes. Clustering was performed on expression profiles of 1311 down-regulated genes, which fell into three groups. The number of genes in each group is given in white, within the dark circles, and the number of transcription factors in each group is given in black, below the circles. **B.** The normalized mean expression profiles for each of the three clusters are shown as graphs, with normalised expression plotted against time. **C.** Transcription factors that fell within the down-regulated clusters are listed.

There were 1311 genes down-regulated by at least 1.5 fold on one or more time points, and we clustered these genes according to their expression patterns by Pearson correlation (at threshold=0.8). This revealed down-regulation in three major waves (Figure 3A–3C). The first wave of down regulation included some genes which have been previously well defined at this transition and are known to be repressed by pre-TCR signalling, and have E2A binding-sites, such as *Notch1, Notch3* and their canonical target *Hes1* [6, 31]. *Runx1,* and the Ets family member *SpiB*, whose forced expression have been shown to block pre-TCR induced differentiation, were also grouped in this expression cluster [32, 33] (Figure 3B). Thus, in the first wave of down-regulation, there is rapid reduction in expression of regulatory genes (>50 transcription factors), including genes that must be down-regulated to allow pre-TCR induced differentiation, and several genes associated with leukaemia/lymphoma. Down-regulation of *Notch1*, *Notch3* and the canonical Notch target *Hes1*, underlines the importance of suppression of Notch signalling at this developmental transition (Figure 3C). The second wave of downregulation included the Wnt-responsive transcription factor Hnf1 (Tcf1), with a well characterised role in T cell development and T-ALL [34, 35], and Smad7, also involved in T-ALL [36], whereas the third wave contained genes that have been less extensively studied in thymocytes.

### Measurement of degradation of transcripts following pre-TCR signal transduction

In order to understand correctly the kinetics of transcription of up-regulated genes, it is necessary to take into account both degradation and accumulation of transcripts [37, 13]. We decided to use a previously validated Genome Wide Transcriptional Modelling (GWTM) strategy to model the kinetics of transcription of up-regulated genes. GWTM has been previously validated experimentally in the transcriptional response of a cell line to irradiation, but not to our knowledge used previously to investigate a physiological developmental process. For this analysis, we selected genes whose expression was up-regulated by >50% on at least one time point, and was not significantly down-regulated during the time course: 3375 genes met these criteria.

**Figure 4:**
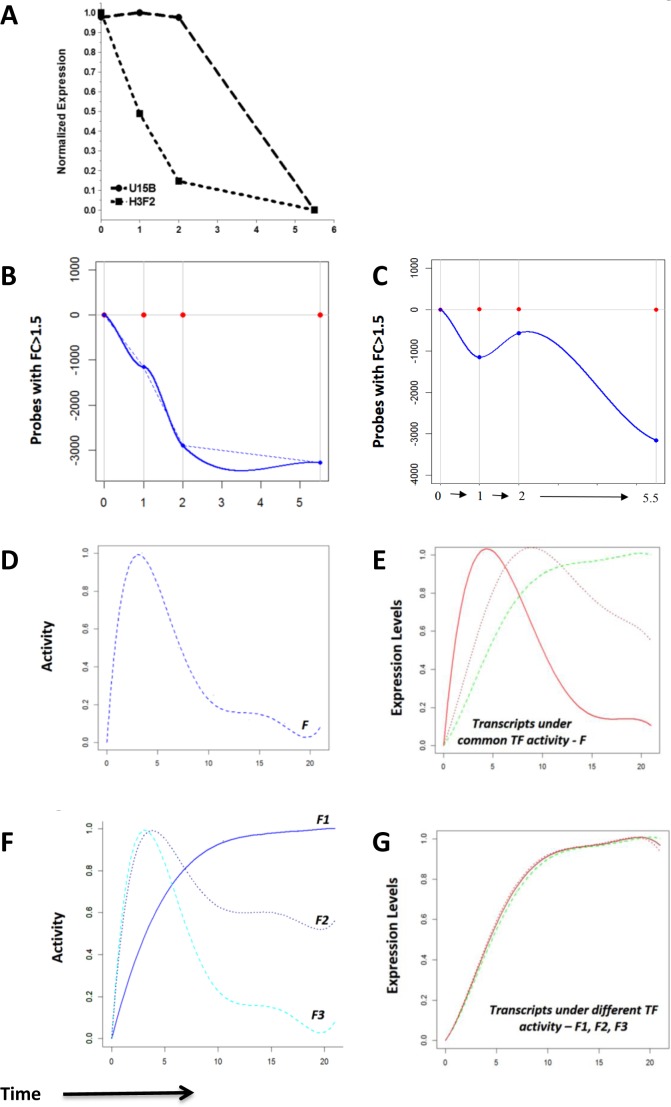
Measurement of transcript degradation and influence of degradation on interpretation of expression data **A.**-**C.** Genome-wide RNA degradation was measured by microarray at stated time points after addition of Actinomycin D to Rag1−/− FTOC after 10 hours of anti-CD3 stimulation. Time zero represents the time of addition of Actinomycin D, and time is shown on the x-axis in all plots. **A.** The decay pattern of transcripts U15b and H3f2 through time in the degradation dataset. **B.** The number of transcripts with fold change >1.5 with respect to time zero in the degradation dataset, shown in the negative part of the x-axis to graphically illustrate down-regulation. **C.** The number of probes with fold change >1.5 at transitions between successive measurements in the degradation dataset, shown in the negative part of the x-axis to graphically illustrate down-regulation. **D.**-**G.** The hypothetical effects of transcript degradation on modelling transcriptional activities, illustrated graphically. **D.** The activity profile, *F*, of a hypothetical transcription factor with **E.** the corresponding normalized responses of three hypothetical target gene transcripts (green, brown and red) with increasing degradation rates. At a higher degradation rate (0.95 for red), the initial response and the subsequent decay are quick such that the transcript expression profile closely resembles the profile of the activity it is driven under. In contrast, transcripts with smaller rates of degradation (0.02 for green, 0.15 for brown) show expression profiles that are delayed. **F.** Three different hypothetical activity profiles, *F1*, *F2 and F3* of different transcription factors with **G.** the expression profiles of their hypothetical corresponding targets, in red, brown and green respectively. The variations in degradation rates of these transcripts (0.95 for red, 0.15 for brown, and 0.02 for green) influence the shapes of their net expression profiles such that all the shapes are highly correlated with each. This can be misleading in cases of clustering solely on expression, and can lead to the false assumption that the transcripts are under one common transcriptional activity.

We first measured genome-wide degradation profiles, by treatment of anti-CD3 stimulated Rag1−/− FTOCs with Actinomycin D to arrest transcription at 10 hours after anti-CD3 treatment. Global gene expression was measured by microarray on addition of Actinomycin D (t=0), through time. Degradation profiles varied greatly among the individual up-regulated transcripts, with some showing no decay until two hours after blocking, and others degrading more than 50% within the first hour (Figure 4A–4C). For example, *H3f2* degraded by more than half in the first hour, whereas *U15b* showed no decay in the first two hours (Figure 4A). Fold change analysis of the decay of 3375 transcripts up-regulated during the expression time-course revealed that 1153 (or 34%) of these rapidly showed 1.5 fold decay in the first hour on the addition of Actinomycin D and 572 of these showed a further decrease by another 1.5 folds in the next hour (Figure 4B–4C). By 5.5 hours, 3275 (or 97%) of the 3375 up-regulated transcripts decayed by 1.5 fold. We incorporated these differences in the rate of degradation of transcripts in the GWTM model to dissect the transcriptional activities downstream of the pre-TCR.

### Genome wide transcriptional modelling

**Figure 5:**
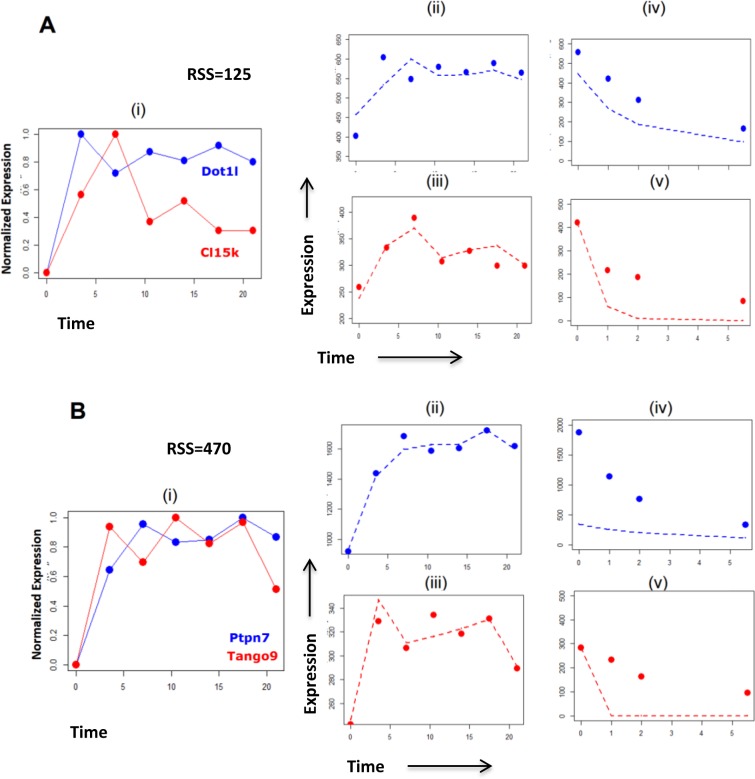
Visualization of the effects of rate of RNA degradation on modelling transcriptional activites, giving examples of specific genes **A.** Known targets of transcription factor E2F4 activity, *Dot1l* and *Cl15k*, display expression profiles (i) which have low correlation. The consideration of the expression data (ii, iii) with the degradation data (iv,v) of both genes simultaneously by GWTM shows a relatively low RSS of 125. This suggests that it is likely that both genes are driven by the same transcriptional activity, since *Cl15k* shows a much higher degradation rate than *Dot1l*, leading to the differences in their expression profiles. **B.** Two transcripts *Ptpn7* and *Tango9* showed highly correlated expression profiles (i). However, on consideration of their expression (ii, iii) and degradation (iv, v) profiles simultaneously by GWTM, it is clear that both transcripts are unlikely to be driven under the same transcriptional activity as the pair has a high RSS of 470. Thus, the high rate of degradation of *Ptpn7* compared to *Tango9*, makes it unlikely that both genes will fall under the same transcription regulatory activity, despite displaying similar expression profiles. Dashed lines represent the corresponding values obtained by GWTM using optimized parameters for expression and degradation.

Exploration of the effects of degradation on network modelling, using the pre-validated Genome Wide Transcriptional Modelling (GWTM) approach, revealed that the targets of the same transcriptional activity can generate very different expression profiles (Figure 4D–4E). Alternatively, significantly different transcriptional activities may lead to an indistinguishable expression pattern of the targets (Figure 4F–4G), misleading us to believe that they are controlled by the same transcriptional activity. For example, the E2F4 targets *Dot1l* and *Cl15k*[38] showed expression profiles with low correlation, but GWTM modelling correctly accommodates the effect of the rapid degradation rate of *Cl15k* to group both genes together under the same transcriptional activity (Figure 5A). Visualization of the model fitting is shown in Figure 5A. In contrast, despite being very well correlated in expression profile, *Ptpn7* and *Tango9*, cannot be driven by the same activity, given the differences in their degradation rates (Figure 5B).

**Figure 6:**
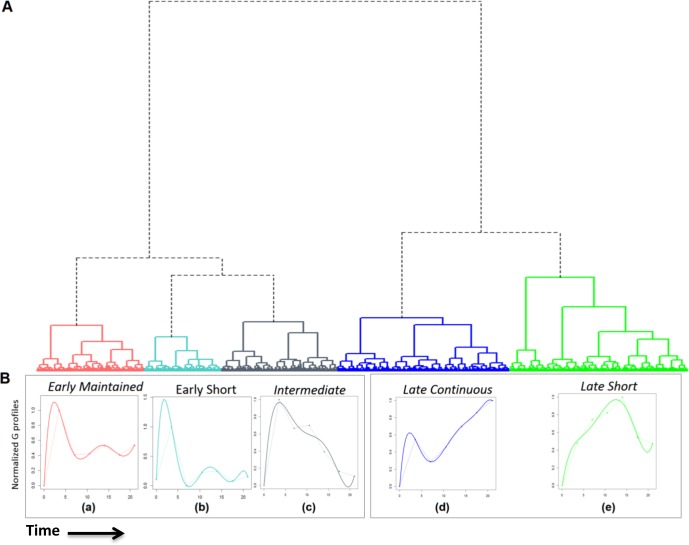
Genome wide transcriptional modelling reveals five principal groups of genes that are up regulated by pre-TCR signal transduction **A.** There are five principal transcriptional activities downstream of the pre-TCR signal. RSS were calculated, followed by hierarchical clustering by Ward's minimum variance method using square root RSS as the distance measure. The resulting cluster dendrogram is composed of five principal transcriptional clusters (a-e) obtained under two major transcriptional responses. **B.** Compound production values, G, were calculated for all genes. The normalized mean G profiles for each of the five clusters are shown to visualise the transcriptional activity profile. Dotted lines join the calculated points and coloured lines show the best fitting curve.

Simultaneous consideration of the differences in the expression and degradation of the transcripts using GWTM[13] thus allowed us to dissect the transcriptional activities downstream of the pre-TCR and to cluster the up-regulated genes according to the shared transcriptional activities that control them, in order to place them in a dynamic temporal context. To separate transcriptional clusters, we determined residual sum of squares (RSS) between all possible 5693625 pairs of the 3375 genes and then applied hierarchical clustering by Ward's minimum variance criterion (Figure 6A). We identified two major transcriptional responses, the first controlled by a transcriptional activity that peaked immediately after the pre-TCR signal and the second by a regulatory activity that built up slowly and peaked later. These transcriptional phases gave rise to five principal transcriptional activities downstream of the pre-TCR signal, based on the decay kinetics of regulatory activity. To visualise the dynamics of transcription in these principal activity clusters, we calculated for each of the five clusters, the mean production profile (G profile), which represents an afﬁne transformation of the active transcription regulator activity controlling the genes (Figure 6B). These profiles (Figure 6B) thus represent the average pattern of transcription through time of the genes in each of the five clusters. The genes in each of these activity clusters are provided in Supplementary Tables 1-5. Consistent with the observation of a second gene flux in the second half of the time-course, we found that 85% of the transcripts newly up-regulated greater than 2-fold at 14.5 hours (Figure 1D) were under the control of the slower transcriptional activities (late continuous and late short).

### Identiﬁcation of transcription factors in the five principal clusters

**Figure 7:**
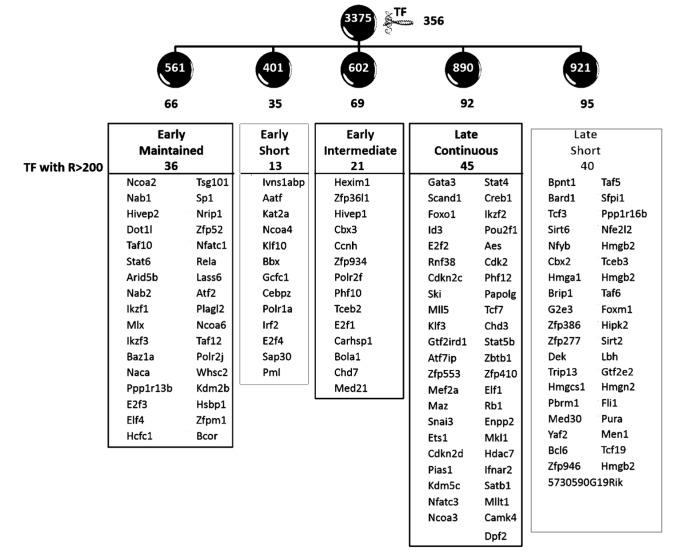
Transcription factors affected by the principal transcriptional activities The figure shows that the 3375 transcripts modelled using GWTM clustered into five clusters of 561, 401, 602, 890 and 921 genes, named ‘early maintained’, ‘early short’, ‘early intermediate’, ‘late continuous’ and ‘late short’ clusters respectively. Within each of these clusters controlled by the five principal transcriptional activities were transcription factors (TFs), the numbers for which are shown in black. The transcription factors varying with a range (R) >200 units (non-log RMA) in each of the five clusters are shown.

We found that 356 of the up-regulated genes encoded transcription factors (>10%), which were divided as 66, 35, 69, 92 and 95 within the five activity clusters, and those with range greater than 200 units (RMA normalized non-log values) are shown in Figure 7. A large number of these transcription factors (>50) have not to our knowledge been previously associated with any specific function in lymphocytes, or T cell development. Among those previously characterized in lymphocytes, several regulators such as the *Nfat*s, *Gata3* and *Id3* are already known to influence pre-TCR induced development [6, 9, 39]. For these factors, the transcriptional model is useful to reconcile the existing literature with a temporal perspective. For example, Nfat is known to be downstream of pre-TCR signalling and involved in early thymocyte development [40–42]. The model revealed that the pre-TCR signal first triggers the principal transcriptional activity controlling Nfat isoform *Nfatc1*, and maintains it throughout the transition. In contrast, the isoform *Nfatc3*, is controlled by a late activity which builds steadily over time, similar to that of *Gata3* and *Id3*. This is consistent with the recently observed effects of short-term mRNA knockdown of different Nfat isoforms on peripheral T cell activation [43], suggesting specific roles of the isoforms through differential regulatory activities. Several of the transcription factors controlled by the various activities are involved in patho-biology of leukaemia and lymphomas. For example, transcription factors Dot1l and Nrip1 which are part of the ‘early-maintained’ response have been implicated in mixed lineage leukaemia [44] and acute lympoblastic leukaemia [45] respectively.

**Figure 8:**
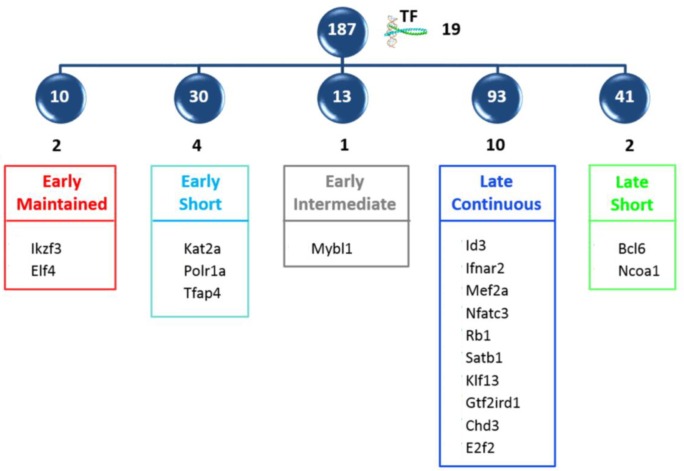
Intersection of modelled transcription factors with high scoring CCA genes The intersection of the 356 transcription factors with the top 1000 genes of the expression dataset contributing to the DN3->DP CCA score, revealed 19 important transcription factors, which are shown distributed between the five principle clusters.

In order to select novel transcription factors, important for differentiation from DN3 to DP, the 356 transcription factors were intersected with the 1000 genes from the expression dataset, which contributed most to the DN3→DP scores in the CCA analysis. This intersection high-lighted 19 transcription factors, distributed between the five major clusters, that are likely to be important at the transition (Figure 8). These could be divided into three functional sets: (a) those with generic roles in cellular differentiation, such as the modifiers of chromatin structure Chd3 and Satb1 [46, 47]; Polr1a [48], the catalytic unit of RNA polymerase, and Rb1, the regulator of cell cycle; (b) those known to regulate T cell development in the thymus, such as Klf13 [49], Id3 [6], Nfatc3 [39]; and (c) those with defined roles in differentiation of cells outside the thymus but still uncharacterized in thymocyte development.

## DISCUSSION

This time series analysis of the transcriptional changes induced by pre-TCR signalling in a synchronised population of developing thymocytes in their thymic environment uncovered a complex transcriptional response in the first 21 hours following the stimulus, at time points when cell surface changes were not yet apparent. The analysis revealed many transcription factors that have not previously been investigated in thymocyte development to be transcriptionally regulated by pre-TCR signal transduction. Intersection of these factors with genes that contribute highly to the DN3→DP CCA scores high-lighted genes that will be candidates for future functional studies. Analysis of down-regulated transcripts revealed that down regulation of transcription following pre-TCR signalling occurs in three main waves.

Our study measured genome-wide RNA degradation of individual genes and found great heterogeneity in rates of RNA degradation of individual genes. This finding underscores the importance of examining both expression and degradation profiles in investigating up-regulation of transcription in response to a stimulus. GWTM has previously been validated in experiments to test the response of a cell-line to irradiation. Here, we applied GWTM to a biological system, and refined the methodology to take into account both expression and degradation in a single step, thus improving fine-clustering (see Supplementary Information).

Our GWTM model is useful to place both known regulators of this stage of differentiation and genes that have previously been shown to be involved in T-ALL, in a temporal perspective. The model revealed five main transcriptional activities downstream of pre-TCR signalling, and in the future it will be interesting and important to identify the transcriptional regulators that control these main activities.

In addition, our time-series expression and degradation data will provide a valuable publically available resource to the research community, which complements microarray datasets from Facs sorted developmentally defined thymocyte populations.

The meaningful implementation of GWTM in response to the pre-TCR signal required us to measure the transcriptome in a synchronized population of cells. We therefore used the Rag1−/− FTOC+ anti-CD3 system, as it allowed thymocytes to remain in their normal cellular environment, but to receive the pre-TCR signal simultaneously. This culture system has been used for many years to investigate pre-TCR induced development from DN to DP thymocyte, but in order to validate the experimental system, we used CCA to compare the time series transcriptome to those of sorted DN3, DN4 and DP populations from the Immgen database. CCA showed here that the transitions over time in the experimental system mirror the molecular changes on the genomic level in the pre-characterized highly purified T-cell precursors DN3, DN4 and DP, such that the molecular profiles of the stimulated cells came to resemble more and more the profile of the more differentiated cell product population over time. Thus, the transcriptional changes that take place in Rag-deficient thymocytes when treated with anti-CD3 monoclonal antibody mirror those that are measured in sorted developmentally defined thymocyte populations from the adult, validating this experimental system.

In summary, our study highlights the benefits of time course analysis in the investigation of the regulation of differentiation and allows us to visualise the complexity of the transcriptional response to a single developmental stimulus, in a way that would not have been possible from a simple comparison of the transcriptomes of sorted thymocyte populations.

## MATERIALS AND METHODS

### Animals

Rag1−/− mice, purchased from The Jackson Laboratory (Maine, USA), and C57BL/6 were bred and maintained at UCL under UK Home Office regulations.

### FTOC

E17.5 Foetal thymi were cultured on 0.8μm Millipore filters (Millipore, Massachusetts, US) on 1ml AIM-V serum free medium (Invitrogen, US) in 24-well plates for one day and then stimulated with 1μg/ml anti-CD3ε (BD Pharmingen, US). Actinomycin D (Sigma-Aldrich, US) at 10μg/ml was added to the culture wells to arrest transcription where stated.

### Antibodies and flow cytometry

Cell suspensions were prepared and stained using combinations of directly conjugated antibodies supplied by EBioscience (San Diego, US) and analysed using Flowjo 10.6 (Tree Star, US) as described [25].

### Microarray time course with anti-CD3-treated Rag1−/− thymocytes

Thymocytes were extracted by crushing thymus lobes between 2 pieces of ground glass. For expression arrays Rag1−/− FTOCs were used at the time points 0, 3.5, 7, 10.5, 14, 17.5, 21 hours following anti-CD3 stimulation; while for degradation arrays Rag1−/− FTOCs were used at 0, 1, 2, 5.5 hours following Actinomycin D addition into the 10 hour anti-CD3 stimulated cultures. Thy1.2+ cells with a purity of >90% were magnetically separated by positive selection using the EasySep system (Stem Cell Technologies, BC) with DynaMag-PCR-Magnet (Invitrogen, US) following manufacturer's instructions. RNA for microarray analysis was extracted using Arcturus PicoPure RNA Isolation kit (Applied Biosystems, US) following manufacturer's instructions and quantity and quality determined by Nanodrop spectrophotometer and Bioanalyser 2100 (Agilent). Micorarrays were performed by UCL Genomics on the Affymetrix Mouse Gene 1.0 ST Platform (GPL6246) using standard Ambion (Invitrogen, US) chemistry. All microarrays are publically available on ArrayExpress (E-MTAB-3088).

### Transcript level prediction in degradation arrays

An iterative procedure was used to normalize the degradation arrays [13].

### GWTM and the simultaneous consideration of expression and degradation

GWTM considers the rate of expression as well as degradation for identification of transcriptional clusters and in-principle could be applied to both upregulated and downregulated genes. However, in this study, only the upregulated genes have been considered for GWTM. GWTM was not applied to down-regulated genes in this study because in the case of down-regulated genes there is no way to measure and distinguish between the decrease in mRNA due to transcriptional down-regulation on stimulation of the cell from the down-regulation due to rate of degradation.

The GWTM equation can be re-arranged in a discrete vector-time representation to predict the transcript expression:

$$x_{k}\;=\;{\left(A+D_{k}I\right)}^{-1}(B_{k}+S_{k}f)$$

where A is a differential operator approximate. For two transcripts that are co-regulated, all above parameters are specific to the individual transcripts other than the time varying transcriptional factor activity f which is shared. Both the equations predicting transcript concentrations in degradation and in expression arrays were simultaneously considered for a transcript pair and compared with the empirically observed values to calculate a residual function i.e. the squared difference between transcript concentrations predicted by the model equations and the experimentally measured concentrations at each data point divided by variance of the data value. For every transcript pair possible, the transcript specific parameters for each transcript along with the shared f were optimized until the minimal value of the sum of squared residuals was achieved using Levenberg-Marquardt algorithm (LMA). These RSS were used as a measure of distance between all pairs of transcripts to which the model was fitted. This allowed us to take into account uncertainties in empirical degradation rates and allow for these in subsequent stages of the model to deliver a better grouping at sub-cluster level than a two-step approach of first calculating degradation rates empirically, and then using them to calculate compound production for clustering by shape (See Supplementary information for implementation and comparison on an experimentally validated data set).

### First derivative estimation from data

The entries of the differential operator A are obtained by rational functions of the time intervals between observation points, thereby implementing Lagrange interpolation as described [37]. In this study, we arrived at the following matrix, which is applicable to the case of seven equally spaced measurements between 0 and 21 hours.

$$A=\;\left[\begin{array}{lllllll} 0 & 0 & 0 & 0 & 0 & 0 & 0\\ -17/63 & 1/7 & 1/7 & -1/63 & 0 & 0 & 0\\ 31/252 & -8/21 & 1/7 & 8/63 & -1/84 & 0 & 0\\ 0 & 1/42 & -4/21 & 0 & 4/21 & -1/42 & 0\\ 0 & 0 & 1/42 & -4/21 & 0 & 4/21 & -1/42\\ 0 & 0 & 0 & 1/21 & -2/7 & 1/7 & 2/21\\ 0 & 0 & 0 & 0 & 1/7 & -4/7 & 3/7 \end{array}\right]$$

All matrix rows sum up to zero and the first row is filled with zeros as it is assumed that the net rate of change for every transcript is nil at time zero at which the stimulus is applied.

### Clustering and pre-clustering filtering

To select up-regulated genes to analyse, we used transcripts with an RMA normalized expression range of 1.5 or greater, that were up-regulated in at least one time point during the expression time course and were not lower than ten percent of the time zero value at any time. RSS for every possible transcript pair was calculated and agglomerative clustering performed based on the square root of RSS as a distance measure using the hclust clustering function in R. Genes were grouped according to Ward's minimum variance to minimize total within-cluster variance. Transcription factors were identified by intersection with the 1838 murine transcription factors in the Riken Genomics Transcription factor database (version 2013) [50].

### Canonical correspondence analysis

Canonical correspondence analysis on microarray data (CCAM) was carried out as previously described to analyse CD4+ T cell populations in the context of helper T cell subsets and to analyse disease samples in the context of hematopoietic cell differentiation [24–26]. To represent the environmental variables of interest, the top differentially expressed genes (based on p-values) between the respective starting and ending precursor population were used. The experimental time-course microarray samples were then linearly regressed onto the environmental variables. The CCA function of the CRAN package library ‘vegan’ [51] was used for the calculations. Briefly, the CCA first regresses the dataset for experimental samples onto differentiation variables (also called environmental variables) i.e. the gene expression gradient obtained from well characterized datasets. The differentially expressed genes used for the well characterized datasets were obtained using eBayes moderated t-statistics. CCA finds new axes by assigning numerical values to samples and genes in order to maximize the dispersion. This is algebraically equivalent to performing singular value decomposition of the matrix that is standardized in the χ2 metric. In CCA, the weighted variance (or inertia) is equivalent to the eigenvalues of PCA. Selection of genes is fixed if inertias are to be compared between different differentiation variables. However, when comparisons are made between the inertias of different differentiation variables, different number of genes are selected for different variables. Further details on CCA can be read here [52, 53]. To represent the environmental variables of interest, the top two thousand differentially expressed genes (based on p-values) between the respective starting and ending precursor populations were used. The p-values after adjusting for false positives were calculated by moderated eBayes using three different microarray samples deposited in the Immgen Database [27] (Geo: GSE15907) for each of the DN3a, DN4 and DP T-cell precursor populations with >99% purity.

## SUPPLEMENTARY MATERIAL FIGURE AND TABLE












