# Supplementary material for: A genome wide transcriptional model of the complex response to pre-TCR signalling during thymocyte differentiation
Source: Oncotarget. 2015 Sep 22;6(30):28646–60. doi: 10.18632/oncotarget.5796 (PMC4745683; doi:10.18632/oncotarget.5796)
Supplement: Supplementary file 1 [file oncotarget-06-28646-s001.pdf]

A genome wide transcriptional model of the complex response to pre-TCR signalling during thymocyte differentiation

Supplementary Material

Implementation of GWTM

To implement GWTM, the nls.lm function of the minpack.lm library was used. The function needs initial parameter values which are then optimized. In this section, the parameters to be optimized are explained along with any scaling done to them. We then compare the results with or without using replicates, and effects of an alternate step-wise approach on sub-clustering in which degradation rate are first calculated followed by gene clustering based on G profiles.

For any transcript pair, a total of 17 parameters were optimized. 10 out of them were specific to each transcript, while the remaining 7 relating to ***f*** were shared by a pair. Transcript specific parameters were D_k, B_k, x_{ko}, b_k and S_k. We placed an additional constraint on the individual sensitivities of genes such that (S_{k1} . S_{k2} = 1) to ensure that the model fitting converges. When $B_k + S_k f(t)$ i.e. the total production at time t was optimized to be less than zero for any data point, it are appropriately scaled to zero or in case of D_k to 1% of peak transcript concentration in transcription blocked microarray time course. This is since zero cannot be used as the minimum value of D_k, as it leads to (A+DI) quantity to become singular and thus non-invertible because of which fitting cannot be done.

Initial Values for Degradation Parameters

The starting value for the degradation model parameter x_{ko} is provided from the experimental transcript concentrations at time zero in transcription blocked microarray time course arrays. Curvature parameter b_k is considered zero initially

and the equation $x_k(t) = x_{ko}e^{-D_k t}$ is used determine initial transcript concentrations. Initial D_k value is the negative of the gradient of best line fit after natural logs are taken on both sides of the above equation.

Initial Values for Expression Parameters

The system before stimulation is considered at equilibrium. Hence, from the GWTM equation, the initial basal level expression $B_{k0} = D_k x_{k0}$. To estimate the initial values of S_{k1}, S_{k2} and ***f***, we re-arranged GWTM equation to first define another term **E** as $\mathbf{E_k} = S_k \boldsymbol{f} = (A + D_k I)^{-1}\boldsymbol{x_k} - B_k$ .Now, since ***f*** is shared by both transcripts in a pair that is analysed, we divided the sum of absolute values of $\mathbf{E_{k1}}$ and $\mathbf{E_{k2}}$ to determine an estimate of S_{k1} and S_{k2} to use for obtaining initial parameters as under.

$$\frac{\sum_{j=2}^{7}\left|\boldsymbol{E}_{\boldsymbol{k1}_j}\right|}{\sum_{j=2}^{7}\left|\boldsymbol{E}_{\boldsymbol{k2}_j}\right|} = \frac{\sum_{j=2}^{7}\left|S_{k1}\boldsymbol{f}_j\right|}{\sum_{j=2}^{7}\left|S_{k1}\boldsymbol{f}_j\right|} = \frac{S_{k1}}{S_{k2}} = {S_{k1}}^2 \quad => \left(\frac{\sum_{j=2}^{7}\left|\boldsymbol{E}_{\boldsymbol{k1}_j}\right|}{\sum_{j=2}^{7}\left|\boldsymbol{E}_{\boldsymbol{k2}_j}\right|}\right)^{1/2} = S_{k1}$$

Initial S_{k2} was determined as reciprocal of S_{k1} above. The initial value of ***f*** was then calculated as the mean of $\mathbf{E_{k1}}/S_{k1}$ and $\mathbf{E_{k2}}/S_{k2}$**.** If any of the terms in this initial ***f*** was found to be negative and the total production at a time point was calculated less than zero, then the initial ***f*** was made zero by multiplying ***f*** by $-B_k/(S_k f(t)_{min})$ where $f(t)_{min}$ is the most negative element of ***f***.

Effects of Biological Replicates on clustering

We observed the effects of using replicates on clustering using 30 probes randomly chosen from the 200 genes modelled and assigned into validated clusters by Martino et al., 2009. The comparison revealed that in both cases GWTM divided the 30 transcripts in two major transcriptional clusters of strength 18 and 12. Further, out of the 30 genes, 28 were clustered in the same major clusters by both the approaches

with only one incorrectly clustered in each of the two major clusters when using the biological replicates indicating that the model is useful in dissecting the transcriptional response when applied to the research data-set generated in this study.

Table S1 – Effects of replicates on clustering with discrepancies in red. The percentage in brackets represents the coverage by the modelling without biological repeats w.r.t to the modelling with

**Cluster 1**	**Cluster 2**
201236_s_at	201464_x_at
201631_s_at	201466_s_at
201834_at	201502_s_at
202181_at	201739_at
202284_s_at	201791_s_at
202672_s_at	201801_s_at
204780_s_at	202021_x_at
205780_at	202107_s_at
208796_s_at	202643_s_at
209295_at	202644_s_at
212815_at	202687_s_at
213293_s_at	202074_s_at
215719_x_at	**(91.6%)**
216252_x_at	
218007_s_at	
218346_s_at	
218627_at	
201329_s_at	
**(94.4%)**	

Effects of incorporating errors in calculating degradation rates on sub-clustering

To assess the effects of incorporating errors in calculated degradation rates, we clustered 200 transcripts used in Martino et al 2009 by simultaneous consideration of degradation and expression as explained earlier; and by the alternate approach of first calculating degradation rates and using it to cluster genes by G profiles. Both approaches led to three major clusters corresponding to – an early NFkB/c-jun/AP-1response, a delayed response controlled by p53 and a late response related to cell cycle re-entry, all of which were in close agreement (data not shown). However, the transcriptional sub-clusters produced were very different by both approaches. These sub-clusters may be interesting from the point of view of subtle biological activities and may be influenced by different sub-regulators which operate under the same major transcriptional factor activity. The differences in clustering arise because simultaneous consideration allows for uncertainties in measurement of degradation to be

incorporated in subsequent steps unlike in the more rigid step-wise approach. In absence of an absolute standard prescription of transcriptional sub-clusters, we compared the results of clustering by both approaches with functional gene groups generated at each level using DAVID (Database for Annotation, Visualization and Integrated Discovery) – Figure S1. The structure of functional groups remained intact on sub-clustering from one level to next when uncertainties in degradation were incorporated in the model in contrast to the two step model when a fixed calculated degradation rate was used. This indicated simultaneous consideration of expression and degradation approach is better than step-wise use of expression and degradation data in identifying subtle transcriptional activities and sub-regulators; therefore, the former approach is applied to the research data-set generated in this study.

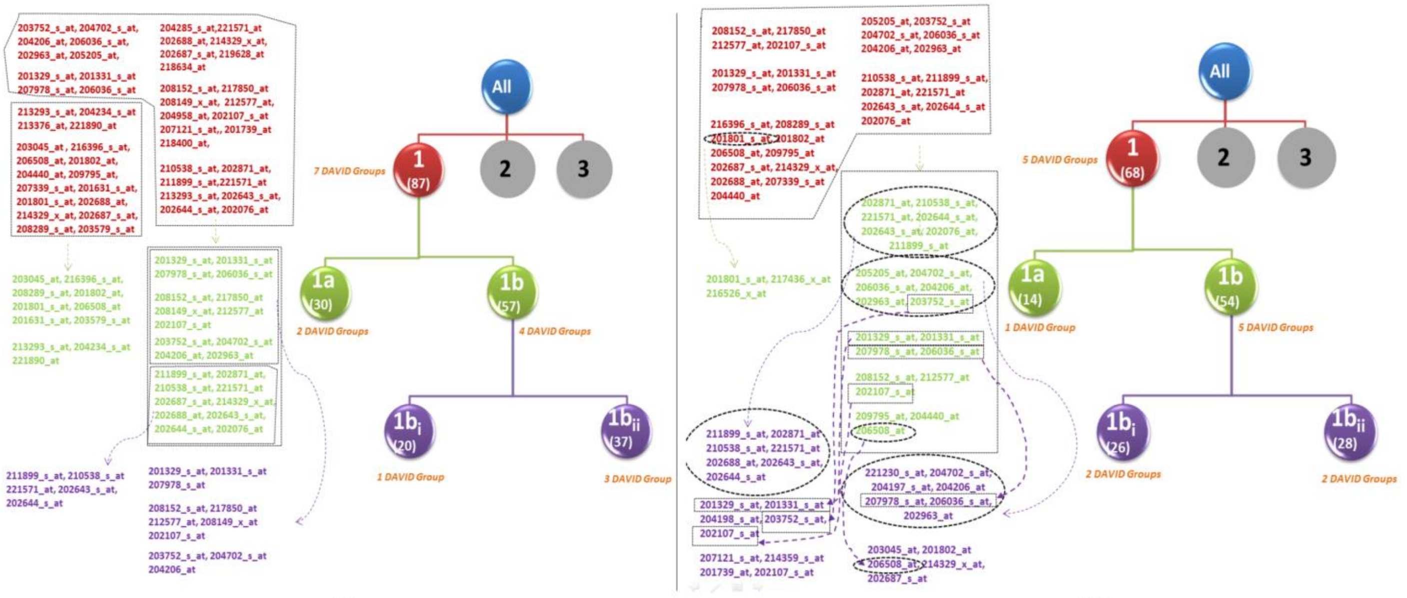

(A) **(B)**

Figure S1 – Effects of incorporating errors in calculating degradation rate on clustering. Figure shows the clusters generated by simultaneous consideration of degradation and expression **(A)** and by the two step approach of first calculating the degradation rate and using to determine G profiles for clustering **(B)**. The most enriched DAVID functional gene groups within the corresponding sub-clusters of major cluster 1 are shown in both cases. Colours indicate the level of sub-clusters and values in bracket are the total number of transcripts in the respective cluster. Dashed arrow show the pattern of sub-grouping from one level to next on the basis of DAVID.
