# Supplementary material for: A genome wide transcriptional model of the complex response to pre-TCR signalling during thymocyte differentiation
Source: Oncotarget. 2015 Sep 22;6(30):28646–60. doi: 10.18632/oncotarget.5796 (PMC4745683; doi:10.18632/oncotarget.5796)
Supplement: Supplementary file 2 [file oncotarget-06-28646-s002.pdf]

Rab23
Zap70
Lipt1
Osgepl1
Fam117b
Rpl17
Pikfyve
Sp100
Inpp5d
Dgkd
Rnpepl1
Atg4b
Rab3gap1
Pik3c2b
Cd84
Slamf9
Exo1
Itpkb
Ncoa2
Lsm5
Rev1
Kdelc1
Nab1
Als2
Sumo1
Kansl1l
Bard1
Csprs
Rpl27a
Swt1
Tor1aip1
Ephx1
Nvl
Mtrf1l
Rmnd1
Nup43
Gm5177
Sf3b5
Hivep2
Trdn
Ccdc138
Micu1
Rpl27a
Lsm7
Dot1l
Zfp873
Taf10
Osbpl8
Stat6
Rps8
Ccdc109a
Arid5b

Mif
Agpat3
Klf16
Lsm7
3200002M19Rik
Thap2
Shmt2
Cspp1
Nab2
Gm129
Sf3a1
Osm
Ikzf1
Xpo1
Rapgef6
Cops3
Ctdnep1
Dlg4
Mir212
Gemin4
Git1
Atad5
Slfn3
Ddx52
Rnft1
Coil
Socs7
Mlx
Tubg2
Cd300a
Ten1
Socs3
Nefh
Etaa1
Commd1
Rpsa
Ccdc69
Gemin5
2310004I24Rik
Kdm6b
Pfn1
Abcc3
1700001P01Rik
Ikzf3
Gpatch8
Gpatch8
Dcakd
Asxl2
Rdh14
Rpl29
Smc6
Rpl36

Gm9292
Prkch
Snapc1
Batf
Spata7
Otub2
Mrto4
Cbll1
Baz1a
2700097O09Rik
Ppp2r3c
Rps29
Pole2
Nemf
Trmt5
Erh
Gm8300
Naca
Cspp1
Galc
Ppp1r13b
Hist1h2bp
Zfp184
Hist1h2ab
Zfp934
Zfp759
Rsl1
Tmem161b
Esco1
Akr1c18
Pfkp
1600012F09Rik
Hist1h2ao
Hist1h2bc
E2f3
Ccdc90a
Nfil3
Il9
Cdc14b
Mterfd1
Zfp708
Zfp953
Zfp456
Zfp874b
Zfp738
Zfp85-rs1
Mtrr
Zfp72
Zfp825
Rfesd
Ankrd32
Xrcc4

Polk
Mrps36
Ppwd1
Zswim6
Slbp
Anxa11
Arhgef3
Btd
Oxnad1
Gapdh
Gjb2
Mtmr6
Lcp1
Uchl3
Gm5458
Zmynd17
Ccdc66
Rpl7a
Gnpnat1
Wdhd1
Cenpj
N6amt2
Ptk2b
Lrch1
Naa16
Wbp4
Prkaa1
Lmbrd2
Rad1
Dap
Pvt1
Tsg101
Pdxp
Pphln1
Pced1b
Sp1
Spef2
Zfat
Arc
Pycrl
Vps28
Tubgcp6
Rpap3
Senp1
Nat15
Mettl22
Yars2
Snap29
Ndufa11
Gng5
Ccdc14
Ndufs5

BC027231
Rps24
Rpl24
Morc3
Ehhadh
Gtf2e1
Tfg
Zfp654
Nrip1
Ltn1
Rnaset2a
Airn
BC002059
Zfp160
Zfp51
Zfp52
3110052M02Rik
3110052M02Rik
3110052M02Rik
Zfp760
Zfp758
Bnip1
Rpl21
Rrp1b
Zfp952
Ndufa7
Znrd1as
Gm9104
Mrps18a
Ccnd3
Ndufa11
Glcci1
Zfp942
Zfp944
Zfp40
Gfer
Bak1
Zfp763
Mrps18b
Rpp21
Rpl11
Apobec2
Smchd1
Hmgb1
Rpl7a
Rab18
Thoc1
Rae1
Rpl11
Rpl19
Rpl29
Rbm27

Cep192
Cep192
Rps25
Gm10548
Dnajc18
Tcof1
Amd1
Cep76
Nfatc1
Gnpnat1
Dpp3
Slc29a2
Rela
Snhg1
A430093F15Rik
Hectd2
Fam178a
Sfxn3
Nolc1
Nt5c2
Nanos1
Sssca1
Pola2
Stxbp3a Up regulated Genes clustered by RSS in Early Maintained
Cd5
Cd6
Psat1
Gm10136
9930021J03Rik
Tctn3
Mxi1
Itih5
Nelf
Edf1
Gpd2
Tank
Lass6
Gorasp2
Olfr1274-ps
Dtwd1
Zfp937
Rbm39
Ttpal
Zswim3
Hax1
Rae1
Rps8
Suv39h2
Mcm10
Fam188a
Atp6v1g1
Gtdc1

Atf2
Lnp
Calcrl
Dgkz
2700007P21Rik
Lrrc57
Zscan29
Rpl27a
Plagl2
Ncoa6
Pdcd10
Rpl37a
Dsn1
Tomm34
Sumo1
Gnas
Rpl29
Mtfr1
Hltf
Rpl27a
Rpl22l1
4932438A13Rik
4932438A13Rik
Smc4
4930579G24Rik
Pdgfc
Sh2d2a
Ubqln4
5830417I10Rik
Pogz
Cdc42se1
Ctsk
Golph3l
Alg14
Gclm
Rpl7a
Syde2
Gng5
Mrpl47
Ndufb5
Cflar
Sclt1
Slc33a1
Snrpd2
Kpna4
Pdcd10
Pip5k1a
Gm129
Rangrf
Sars
Trmt13
Tet2

Rap1gds1
Sh3glb1
Chd7
Chd7
Chd7
Ccne2
Myl6
Chchd2
Nipsnap3b
Trim32
Klhl9
Ifna7
Zcchc11
Slc6a9
Ccdc23
Lsm10
Snord85
Taf12
Gpn2
Gm13154
Gm13034
Mrpl20
Tox
Gm10136
Ccl21a
Msmp
Rpl17
Haus6
Hnrnpa3
Ndufs5
S100pbp
Sesn2
Zfp933
Tmem201
Gm15772
Nupl2
Papd4
Mthfd2l
BC005561
Zfp326
Zfp605
Arpc3
Polr2j
Got2
Orc5
Rpl22l1
5031410I06Rik
Whsc2
Lcorl
Arap2
Ube2n
Cenpc1

Cox18
Zfp951
Kdm2b
Pitpnm2
Aimp2
Tsen15
Gars
0610030E20Rik
AW146020
Mogs
Zfml
Alms1
C87436
8430410A17Rik
Kbtbd8
Zfp637
Rpl7a
Braf
Tarbp2
Ppia
Rpl27a
Wnt5b
Ccdc77
Cd27
Recql
Bcat1
Kras
Rps29
Phf20
Irf2bp1
2310022A10Rik
Pin4
Myod1
Hmgb1
Tm2d3
Mrps11
Fchsd2
Smpd1
Snora23
2610020H08Rik
Eif3c
Rassf7
Zik1
Zbtb45
Pvr
Gm4881
Pgam1
Pld3
Plekhg2
Samd4b
Zfp27
Sdhaf1

Pop4
Ctdnep1
9830147E19Rik
Gm5595
Snord34
Kcnj14
Cib1
Pold3
Nup98
Gm4759
Palb2
Zfp828
Thap1
Rpl29
Dusp4
Vps37a
Mlf1ip
Nek1
Sh3rf1
Nat2
Zfp930
Pgls
Zfp709
Amfr
Pdp2
Dus2l
Cog8
Cmip
Hsbp1
Zfpm1
Rpl13
Plekha2
Ddhd2
Gm8096
Lonrf1
Ccdc111
Cdkn2aip
Gatad2a
2810428I15Rik
Uba52
Rpl23a
Cherp
Lphn1
Ccdc130
Rfwd3
0610007P22Rik
Egln1
Maml2
Bud13
Gm5617
Pou2af1
Npat

Ube2q2
Tipin
Gm7265
Zfp949
Bcl2a1a
Rpl7a
Dbr1
Dusp7
Wdr48
Zfp167
Uba52
Chek1
Gramd1b
Ccdc84
Snx1
Slc25a40
Rasa2
Phf16
Wdr44
2610018G03Rik
Phf6
Taf1
Cox7b
Rragb
A830080D01Rik
Piga
Gemin8
Larp4
Bcor
Elf4
Zfp280c
Fgf13
Haus7
Hcfc1
Zxda
Rps6ka6
Rps12
Zmat1
Tmsb15l
Tspyl2
G530011O06Rik
