# Supplementary material for: A genome wide transcriptional model of the complex response to pre-TCR signalling during thymocyte differentiation
Source: Oncotarget. 2015 Sep 22;6(30):28646–60. doi: 10.18632/oncotarget.5796 (PMC4745683; doi:10.18632/oncotarget.5796)
Supplement: Supplementary file 3 [file oncotarget-06-28646-s003.pdf]

Eif5b Upregulated gene cluster Early Short

Ormdl1
Rpe
Phlpp1
Gm7246
Zbtb41
Ivns1abp
Tor1aip2
Gas5
Gas5
Pyhin1
Mfsd7b
Lpgat1
Arfgef1
Sumo2
Ccdc115
Mitd1
1110034B05Rik
Fam126b
Snora75
Snord82
Ptpn4
Atp2b4
1700025G04Rik
Rgl1
Cacybp
Suco
Ddr2
Opn3
Hace1
Ascc3
Ranbp2
Tmem26
Slc19a1
Gadd45b
Eea1
Atp2b1
Ccdc59
Rpl29
Ginm1
Utrn
Ltv1
3110003A17Rik
4930444G20Rik
L3mbtl3
Trmt11
Ncoa7
Qrsl1
Ddx21
Rpl23a
Utp20
Tbk1

Morc2a
Zmiz2
Fbxo48
Fancl
Pnpt1
Fndc9
Zcchc10
Fam18b
1300001I01Rik
Mettl16
Adap2
Slfn2
Car4
Rpl13
Sec14l1
Baiap2
Rel
Rars
9930111J21Rik2
Cd68
Dhx33
Slfn9
Aatf
Snora21
Ccr7
Atp5g1
Kat2a
Tex2
Nol10
Cog5
Fancm
Mgat2
Prkch
Ncoa4
Ubxn2a
Dus4l
Rpl29
Ankrd9
Dip2c
Heatr1
Tcrg-V4
Tdp2
Riok1
Iars
Erap1
Homer1
Dimt1
Ercc8
Arl15
BC016423
Ryr2
Rpp40

Ankrd32
Rps24
Ktn1
Pspc1
Zc3h13
Mir20a
Gm5796
Zfp503
Gnl3
Gch1
Snord8
Gjb2
Gm6907
Mir15a
Rbm26
Abcc4
Polr2k
Adck5
Apol7e
Tmed2
Prkag1
Larp4
Fbxo4
Ptger4
Spef2
Dnajc21
Brix1
Acot10
Klf10
Ext1
Nhp2l1
Snora34
Ddn
Il1rap
Btla
Ift57
Tfap4
Parn
Alg3
Qtrtd1
Cd200
Abhd10
Bbx
4930453N24Rik
Ltn1
Ltn1
Rpl13
Urb1
Gcfc1
Agpat4
Airn
Zfp948

Zfp943
Uhrf1bp1
Tap1
Mmp25
Tbl3
Zfp81
Prrc2a
Aif1
Tnf
Scoc
Slc29a1
Foxp4
Rab12
Cebpz
Sos1
Slc8a1
Thada
Lrpprc
Cul2
9430020K01Rik
Svil
Taf4b
Gapdh
Rpl35
Hars
Ppargc1b
8030462N17Rik
Socs6
Snhg1
Snhg1
Snhg1
Wdr74
Pdcd1lg2
Il33
Pten
Btaf1
Zfp518a
Fam178a
Pprc1
Cst6
Ctsw
Ccdc86
U05342
Cep78
Cbwd1
Noc3l
Sorbs1
Rrp12
Cwf19l1
Pdcd11
Gm13375
Pdss1

Set
Tnfaip6
Ssfa2
Timm10
Zc3h6
Snord57
Nsfl1c
Tgif2
Rbm17
Dnajc1
Rbm43
Ttc30a1
Ccdc141
Creb3l1
Fam82a2
Slc30a4
Il1a
Slc23a2
Esf1
Kif16b
Zfp120
Zfp442
Cdk5rap1
5730471H19Rik
2210418O10Rik
Rps8
Rsrc1
1110032F04Rik
Tmem154
Mef2d
Prkab2
Igsf3
Dnttip2
4930422G04Rik
4930422G04Rik
Odf2l
Pigk
Hps3
Rnu73b
Rpl21
Gpr89
Hmgb1
Rnpc3
Snhg8
Clca2
Tgs1
Chd7
Chd7
Rpl13
Zcchc7
Zcchc7
E230008N13Rik

Tmem38b
Hook1
Efcab7
Csf3r
Phc2
Gm13139
Nol6
Sigmar1
Rmrp
Olfr267
Ctps
Clic4
Mrto4
Spsb1
Abcb1b
Galnt11
Lyar
Sep-11
Mrps17
Pex1
Nop14
D5Ertd579e
Slain2
Cspp1
Sgcb
Clock
Ppat
Adamts3
Adamts3
Sdad1
Hfm1
AB010352
Noc4l
Mepce
Pilra
Slc7a1
Polr1a
Smyd5
Alms1
Trnt1
Irak2
Zfand4
M6pr
Parp11
Tfec
AB041803
Dfna5
Ptcd3
Chchd4
Zfyve20
Snora7a
Olr1

Ergic2
Vmn2r43
Vmn1r114
Atp10a
Vps33b
Eftud1
Tacc2
Relb
Erf
Nudt19
AI987944
Snord35b
Snord35a
Snord33
Snord32a
Fancf
Mphosph10
Snora21
Tmem135
Snord15b
Snord15a
Gm8995
Rps4y2
Rfwd3
Rpl7a
Aida
Zfp958
Efha2
Irf2
Naf1
Hsh2d
Inpp4b
Herpud1
Nlrc5
E2f4
Snord68
Polb
AY512931
Rnu2-10
Sap30
Gm10033
4930467E23Rik
Neto2
N4bp1
Sp110
Yipf2
Snord14d
Snord14e
Idh3a
Sema7a
Myo9a
Myo9a

Myo9a
Myo9a
Myo9a
Myo9a
Dennd4a
Dennd4a
Dennd4a
Dennd4a
Gm8560
Azi2
Golga4
Zfp558
Pml
Phip
Dnajc13
Dnajc13
Dnajc13
Dnajc13
Atp2c1
Ube2a
Utp14a
Rbmx2
Styx
Xlr3b
Snora70
Tab3
Ar
AV320801
Tbc1d8b
Nudt10
Rnu2-10
Xlr
Mtap7d3
Snord61
Taf9b
Cdkl5
Ofd1
