# Supplementary material for: A genome wide transcriptional model of the complex response to pre-TCR signalling during thymocyte differentiation
Source: Oncotarget. 2015 Sep 22;6(30):28646–60. doi: 10.18632/oncotarget.5796 (PMC4745683; doi:10.18632/oncotarget.5796)
Supplement: Supplementary file 4 [file oncotarget-06-28646-s004.pdf]

Il1rl2 Gene cluster Intermediate

Abi2
Nrp2
Snora41
Rbm44
Fam72a
Ube2t
Gas5
Snord47
Sh2d1b2
Fcgr4
Rnu1b1
Cnih4
Mybl1
Cops5
Lactb2
Gm5523
Slc40a1
Inpp1
Pms1
Rnf25
Sp140
Myeov2
Thap4
Ppp1r15b
Uqcr11
Rgs4
Fcgr3
Slamf8
Gm5069
Mosc2
Fbxo30
Map3k5
Fam54a
Echdc1
Trdn
Trdn
Lilrb4
P4ha1
Icosl
Cstb
Rps15
Oaz1
Thop1
Psma5
Uhrf1bp1l
Usp44
Ccdc41
B4galnt1
Ddit3
Rab32
Arg1

Srgn
Ado
Rnf126
Mars
8430429K09Rik
Aebp1
Ppp3r1
Ccdc88a
Sar1b
Mrpl55
Dhrs7b
Snord49b
Snord49a
Rpl26
Lsmd1
1810027O10Rik
Eno3
Myo1c
Rnmtl1
Dhrs13
Nos2
Ccl11
Ccl8
Wfdc17
Ptrh2
Rnu3b1
Tob1
Cdk12
Erbb2
Grb7
Vps25
Hexim1
Uqcr10
Pold2
Ddx56
Sec61g
Slc1a4
Rps27a
Stc2
Bod1
Sft2d1
Fam183b
Zfp39
Cxcl16
Lgals9
Ccl9
Ccl6
Ormdl3
Psmc3ip
Brca1
Rnu2-10
Trim47

Stra13
Sectm1a
E2f6
Lrr1
Syne2
Gphn
Mpp5
Coq6
Rps25
Snord118
Rnf144a
Ifrd1
Mettl21d
Rdh11
Zfp36l1
0610007P14Rik
Ifi27l2a
4831426I19Rik
Ero1lb
Isca1
Hist1h2br
Hist1h2af
Serpinb9
Hivep1
2010001K21Rik
4933434E20Rik
Cbx3
Stoml2
Glrx
Ccnh
Dhfr
Cenpk
Elovl7
Ppap2a
Gmnn
Gapdh
Zfp934
Cbx3
Cep72
Sec61b
Naip2
Marveld2
Ndufaf2
Parp8
Bnip3
Ptger2
Lgals3
Peli2
Rem2
Mcpt2
Nufip1
Ccdc122

Dnase1l3
Kcnk5
Ero1l
Map1lc3b
Rps12
Ppp3cc
Rpl36a
Lifr
Nup155
Mtbp
Wdr67
Polr2f
Mgat3
Adsl
Rnu12
Dnajc22
Sec61b
Map3k12
Spef2
Rxfp3
Tars
Fbxl7
Ftl2
Nudcd1
Dscc1
Mrpl13
Ly6d
Top1mt
Oplah
Arhgap39
Apol7b
Dmc1
Npcd
D730005E14Rik
St13
Cyb5r3
Rpl39l
Rimbp3
Map3k13
Tctex1d2
Pcyt1a
Rpl35a
Nmral1
Carhsp1
Gspt1
Cdc45
Rps21
Ndufb4
Dzip3
Tmem45a
Hspa13
Btg3

Ltn1
Ltn1
Ltn1
Donson
Zfp677
Zfp229
Pkmyt1
Snrpc
Zfp472
Wdr46
H2-DMa
Fkbpl
Rdbp
Ltb
H2-D1
H2-Q6
H2-Q7
Lsm5
Ubd
H2-M3
Mrpl14
Usp49
Rps24
Lclat1
Phf10
Tceb2
1600002H07Rik
2900010M23Rik
Snord52
Lst1
H2-T23
Ppp1r11
Cd2ap
Enpp4
Mad2l1bp
Bysl
D17Wsu104e
Ticam1
Tceb2
Map4k3
Sft2d1
Rbbp8
Cables1
Vaultrc5
C330007P06Rik
Slc12a2
Malt1
Bloc1s1
Yipf5
Eif3j
Lox
Ppic

Tshz1
Yif1a
Pla2g16
Snord22
Rab3il1
Ms4a4c
D030056L22Rik
Fas
Rpp30
Pcgf5
Hells
Gapdh
Entpd1
Ubtd1
Pi4k2a
Casp7
Ndufs8
Gstp1
Cdc42ep2
Fkbp2
Fen1
AW112010
Ms4a7
Pip5k1b
Aldh18a1
Usmg5
Dclre1a
Kin
Ptges2
Arpc5l
Itga6
Ak4
Mrpl18
Acp2
Rad51
Chac1
Sirpa
Mcm8
Cst7
Dynlrb1
Src
Mybl2
Pigt
Rpp38
Pfkfb3
Ptges
1110008P14Rik
Stom
Idi1
Acvr1
Nr1h3
Mettl15

Cep152
Atp8b4
Fam110a
E2f1
Tmem189
Atp9a
Bmp7
Atp5e
Psma7
Rprl2
Zfp639
Mrpl47
Hspa4l
Larp1b
Higd1a
Kcnn3
Adora3
Ssx2ip
Carhsp1
Efna1
Hax1
Bola1
Olfml3
Dapp1
Clca5
6330407A03Rik
Chd7
Chd7
Chd7
Chd7
Chd7
Chd7
Chd7
Chd7
Chd7
Chd7
Chd7
Chd7
Chd7
Chd7
Chd7
Chd7
Chd7
Chd7
Chd7
Chd7
Chd7
Chd7
Gem
Nbn
Ccnc
Mms22l

Rplp1
Ube2j1
Car9
Glipr2
E230008N13Rik
Slc31a2
Zfp618
Tlr4
Leprot
Tmem48
Orc1
Slc2a1
Mir697
Sdc3
Tmco4
NA
Gm3579
Hmgb1
Gm13154
Ndufaf6
Fam92a
Aqp3
Kif24
Ccl27a
Ccl19
Mrpl50
Rnu1b1
Actl7b
Gapdh
Aldoart1
Rpl34
Mast2
Toe1
Tinagl1
Snora73a
C1qb
C1qc
C1qa
Trappc2
Prdm16
Pion
Klhl7
4930471M23Rik
Rps29
Cd38
Slc34a2
Fam114a1
Pdgfra
Lrrc8d
Idua
Pop5
Zcwpw1

Stag3
Rundc3b
Gm10471
Rbks
Slbp
Gpr125
Bend4
Uba6
Adamts3
Adamts3
Adamts3
Adamts3
Cxcl9
Hmgb1
Tmed5
Atp5k
Niacr1
Syna
Znhit1
Pcolce
Hsph1
Ccdc132
Irf5
Klhdc10
1110001J03Rik
Rny3
Gimap1
Rps15
Tnip3
Ppp1r2
Arl8b
Thumpd3
Rps15
Clec4a1
Clec4d
C1ra
C1rb
Mlf2
Med21
Nacc1
Asns
Fam115c
Rny1
Rarres2
Il23r
Rprl1
Rpia
Hk2
Htra2
Mthfd2
Snrnp27
Antxr1

Eogt
Csgalnact2
Slc2a3
Lrrc23
Rad51ap1
Klrb1c
Plbd1
Gys2
Zscan22
Pla2g4c
Lypd3
Ceacam10
Eid2
Capn12
Zfp382
Krtdap
Fam103a1
Arrb1
Wee1
Adm
Nucb2
Bola2
Gdpd3
Prr14
Plekha1
Bccip
Ptpre
Cd151
Hspbp1
2810047C21Rik1
Bcl3
Axl
Josd1
Haus5
Ffar2
Cebpg
Plekhf1
Gapdh
Mctp2
Anpep
Furin
4632434I11Rik
Gm5037
Hmgb1
P2ry6
Taf10
Cyp2r1
Nupr1
Nsmce4a
Bnip3
F10
Mrps31

Eif4ebp1
Tusc3
4933411K20Rik
Zfp617
Hmox1
Naa20
Zfp827
Ndufb7
4930432K21Rik
Dnase2a
Cdh3
Cdt1
Timm44
Gm9457
Hook3
Ankrd37
Snx25
Gm12070
Eif2s2
Sc4mol
Ssbp4
Gapdh
Smad1
Rln3
Vps35
Gapdh
Nae1
Gcsh
Fam38a
Slc36a4
Chordc1
Pin1
Rpp25
Myo9a
Myo9a
Myo9a
Myo9a
Myo1e
Plod2
Atr
Prkar2a
Ctdspl
Snora62
St14
Atp5l
Gm6981
Atm
Bbs4
Mb21d1
2410127L17Rik
Fam46a
Acpl2

Dnajc13
Rps27
Tfe3
Timm17b
Lonrf3
Pgrmc1
Atp5l
1110012L19Rik
Sec61g
Armcx3
Bhlhb9
Wbp5
Alg13
Cybb
Med14
1810037I17Rik
Zdhhc9
Prrg1
Pdk3
Slc7a3
Xkrx
Gla
Acsl4
Mospd2
LOC100041256
Mrps10
H2-T9
H2-T10
Dynll1
LOC100044193
