# Supplementary material for: A genome wide transcriptional model of the complex response to pre-TCR signalling during thymocyte differentiation
Source: Oncotarget. 2015 Sep 22;6(30):28646–60. doi: 10.18632/oncotarget.5796 (PMC4745683; doi:10.18632/oncotarget.5796)
Supplement: Supplementary file 5 [file oncotarget-06-28646-s005.pdf]

Rb1cc1 Gene cluster Late Continuous
AI597479
Stat4
Nbeal1
Creb1
Smarcal1
Ctdsp1
Heatr7b1
D2hgdh
Mybph
Klhl12
Rpl29
Ptpn7
Kif21b
Mir181b-1
Fam129a
Arpc5
Nme7
Cd247
Fam36a
Tmem63a
Dusp10
Tmem206
Ube2w
Ankrd23
Dnahc7b
Gtf3c3
Gm6810
Ankrd44
Ppil3
Trak2
Ikzf2
A630001G21Rik
Dbi
Ikbke
Gpr25
Ivns1abp
Cep350
Cep350
Dars2
Gorab
Pou2f1
Atf6
Vangl2
Cep170
Fbxo28
Mfsd7b
Nenf
Traf5
3110009E18Rik
Sesn1
Ostm1

Scml4
Tmem194b
Gcc2
Etv5
Pcnt
Itgb2
Cnn2
Midn
Aes
BC025920
Tcp11l2
Igf1
Bbs10
Phlda1
Avpr1a
Mip
Cspp1
Tespa1
Ccdc28a
Gopc
D630037F22Rik
Eif4ebp2
Ccar1
Tet1
Adarb1
4930404N11Rik
Tle6
Nbeal1
Zdhhc17
4930455F23Rik
Frs2
Lyz2
Lyz1
Helb
Irak3
Pan2
Nabp2
Esyt1
Rps26
Cdk2
Dgka
Pisd-ps1
Ccm2
4930415F15Rik
Asb3
Stk10
Cspp1
Lcp2
Zfp62
Grap
Mmgt2
Trappc1

Kdm6b
Arrb2
4930563E22Rik
Atp2a3
1700016K19Rik
Blmh
Ssh2
Phf12
Tubd1
Vezf1
Hoxb3
Skap1
Prr15l
4930523C07Rik
Mllt6
Lasp1
Wipf2
Kif18b
Fmnl1
Axin2
Amz2
Rpl38
Armc7
Patz1
Znrf3
Polm
Myo1g
Papolg
Spnb2
Ccdc99
Itk
Cdk2ap1
Zfp879
Cspp1
Tcf7
Rad50
Ndufs3
Zfp867
Rnf112
Zfp287
Zfp286
Chd3
4933402P03Rik
Med13
Pdk2
Atp5g1
Stat5b
Dusp3
Pyy
Ccdc47
Icam2
Pecam1

Bptf
Rgs9
Gm11711
Alyref
Sirt7
BC017643
Zfp750
Cenpo
1110057K04Rik
Nbas
Nbas
5730507C01Rik
NA
Rrm2
Sypl
Prpf39
Klhdc1
Zbtb1
Exd2
Zfp410
Bdkrb2
Esyt2
1110002L01Rik
3110053B16Rik
Cox7a2l
Pik3cg
Zbed4
Trappc6b
Fbxo33
Sptlc2
Sp4
Tcrg-V1
Hist1h2ap
Hist1h2br
Hist1h2bg
Hist1h2ao
Hist1h2bc
Hist1h2aa
Ubxn2a
Fam120aos
B4galt7
0610007P08Rik
Rpl37a
Tmem167
Rps23
Gcnt4
Trim23
Pde4d
Il6st
Emb
4833420G17Rik
Ptbp1

Akr1e1
Actn2
Hist1h2ao
Hist1h3g
Hist1h4i
Hist1h2ao
Hist1h3f
Hist1h2be
Hist1h2ac
Hist1h3f
Hist1h3a
Nedd9
Tbc1d7
Fgd3
Uimc1
Cxcl14
Agtpbp1
Zfp595
Zfp58
Polr3g
Rasa1
Naip6
Rad17
Top2b
Fam116a
Dcp1a
Mustn1
Nt5dc2
Eaf1
Mudeng
Ear6
Mettl17
Trav14d-3-dv8
Trav14-1
Oxa1l
Prss51
Ints9
Bin3
Rcbtb2
Elf1
Oxsm
Ndst2
Colq
Ear1
Ear1
Ddhd1
Exoc5
Mettl3
Dad1
Prmt5
4931414P19Rik
Cdh24

Homez
Zmym5
Lats2
Rb1
Nudt15
Dnajc15
Akap11
Akap11
Pou4f1
Dzip1
Slc15a1
Fyb
Rictor
Mtmr12
Nsmce2
Trib1
Efr3a
Zfp7
Cyth4
Muc19
Ano6
Zfp740
Skp2
Spef2
Spef2
Fam105a
Enpp2
Sla
Gm628
Scrt1
Rpl29
Apol8
Rac2
Mkl1
Odf3b
Hdac7
Prkag1
Lmbr1l
Bcdin3d
Pou6f1
Galnt6
Map3k12
Cluap1
Mkl2
Nde1
Ufd1l
B3gnt5
Dvl3
Gm8095
BC106179
St6gal1
Opa1

Ubxn7
Tfrc
Heg1
Heg1
Hspbap1
Polq
Spice1
Senp7
Ifnar2
Cluap1
Nlrc3
Slx4
5730403B10Rik
Ypel1
2610318N02Rik
Klhl6
Tbccd1
Gp5
Gtpbp8
Plcxd2
Cd96
Ahcy
Tbc1d23
Ltn1
Ltn1
Ltn1
Ltn1
Dnajc28
Fgfr1op
Fam120b
Ccdc64b
0610011F06Rik
Kifc5b
Syngap1
Ubash3a
Ndufv3
A530088E08Rik
Zfp563
AA388235
H2-Oa
Gpsm3
H2-Q2
Ppp1r18
Trem2
Tnfaip8l1
Fndc1
Ntn3
Ndufb10
Cramp1l
1700062I23Rik
Stk38
Rasal3

Zfp870
Zfp101
Btnl5
Rsph9
Polh
Satb1
Pot1b
Mllt1
Gm9374
Atl2
Gm10190
Srbd1
1700011E24Rik
AW554918
Ammecr1l
Map3k2
Camk4
Pou4f3
Arl14epl
Eno1
Ftmt
2610318N02Rik
Cep192
Rnmt
Cyb5
Rpl29
Tmem173
Rax
4930503L19Rik
Adnp2
Pitpnm1
Gm962
Map4k2
Rasgrp2
Trmt112
Snhg1
Fen1
Syt7
Ms4a4b
Ms4a6b
Fam108b
Stambpl1
Dntt
Lztfl1
Add3
Atrnl1
Sipa1
Tigd3
Dpf2
Tmem179b
Rfk
Rpl10

Obfc1
Vax1
Pdzd8
Sfxn4
Prkcq
Fam166a
Clic3
Setx
Spna2
Cep110
Gsn
Gpr21
Arhgap15
Pdk1
Sp9
Itga4
Prdx6b
Eno1
Slc43a1
Rapsn
Dgkz
Syt13
Ttc17
Abtb2
2410042D21Rik
Rtf1
Slc28a2
Gm14085
Ap4e1
Cds2
Rpl29
2210009G21Rik
Gm561
Zfp341
Gm1332
Stk4
Zswim1
Ncoa3
Cstf1
Th1l
Zfp831
Lsm14b
Camk1d
Celf2
Celf2
Gata3
A830007P12Rik
Ssna1
Lhx3
Surf1
Rexo4
Col5a1

Fam78a
Fam125b
Zbtb26
Cytip
Fastkd1
Tlk1
Cerkl
Rasgrp1
Vps39
Cdan1
Myef2
Trpm7
Itpripl1
Ciao1
Zc3h8
Fastkd5
Foxa2
Pxmp4
Ggt7
Scand1
Tti1
Chd6
Rpl29
Gcnt7
Hrh3
Mynn
Skil
4932438A13Rik
4932438A13Rik
4932438A13Rik
4932438A13Rik
4932438A13Rik
Ccrn4l
Foxo1
Mir15b
Serpini1
Mex3a
Nup210l
Nup210l
Rfx5
Hist2h3c1
Hist2h3b
Hist2h2bb
Rsbn1
6530418L21Rik
4933421E11Rik
Kcna3
AI504432
Wdr47
Sass6
Lamtor3
Mcoln3

Mcoln2
Pde7a
P2ry12
1110032F04Rik
Ift80
Trim59
Snord73a
Ntrk1
Ttc24
Dap3
Scnm1-ps
Tnfaip8l2
Gabpb2
Gm128
Rprd2
Prpf3
Mrps21
Hist2h2aa1
Hist2h4
Polr3c
Cd2
A930005H10Rik
Usp53
Chd7
1110037F02Rik
Bach2
Rngtt
Ube2r2
Trmt10b
Rpl29
Ifnz
Hspb11
Yipf1
Gm12824
Ptch2
Mycl1
Trit1
Inpp5b
Ftl2
Fam176b
Pef1
Laptm5
Wasf2
Cnr2
Id3
E2f2
1700013G24Rik
B330016D10Rik
Mcrs1
Fv1
Trmt112
Pank4

Tmem67
Slc35a1
Smu1
Rnf38
Ptpn3
Akna
Ttc39b
Lrrc42
Zyg11b
Kti12
Cdkn2c
C530005A16Rik
Dph2
Szt2
Szt2
Szt2
Zfp691
Mfsd2a
Macf1
Ccdc28b
Epb4.1
Rps6ka1
Pdik1l
Miip
Fbxo44
Ski
Ccnl2
Pex1
Krit1
Slc25a40
4933402N22Rik
Mll5
Fam59b
Fam193a
Sh3bp2
Add1
Klf3
Nipal1
Exoc1
Cep135
Stap1
Cxcl13
D930016D06Rik
Lrrc8c
Pcgf3
Fbrsl1
2410131K14Rik
Ppp1cc
Pptc7
Lrch4
Zkscan1
Zfp157

A430033K04Rik
Heatr2
Gpr146
Foxk1
Pms2
Uspl1
C030048B08Rik
Dmtf1
Phtf2
Rpl31-ps21
Napepld
Fam53a
Gnpda2
Nfxl1
Usp46
Trmt112
Nup54
Ccni
Abcg3
Ankle2
Selplg
Git2
Sppl3
Dynll1
Adam1a
Tctn1
Rsrc2
Mphosph9
Eif2b1
Gtf2ird1
Pom121
Rhbdd2
Card11
Zfp316
Rpl29
Glcci1
Tes
Fam40b
Akr1b10
Rab19
Ephb6
Gimap8
Gimap4
Gimap5
Tril
Cd8b1
Cd8a
Tia1
Fbxl14
Mrpl51
Atf7ip
Glcci1

Zfp800
Rbm28
Ube2h
2010107G12Rik
Cnot4
Slc37a3
Zfp746
Gimap6
Rpl29
Tmsb10
Dguok
Rassf4
Ankrd26
Clstn3
Cd4
Ptms
5930416I19Rik
Clec7a
St8sia1
Klhdc5
Caprin2
Cnot3
Eps8l1
Epn1
Zfp667
2310014L17Rik
Zc3h4
Prkd2
Kcnn4
Zfp574
Zfp60
Rinl
Rasgrp4
Zfp260
Zfp658
Zfp719
Kcna7
Ftl1
Tmem143
Mrgprb13
Tubgcp5
Akap13
Sema4b
D330012F22Rik
Alg8
Rassf10
D430042O09Rik
Sbk1
Zfp553
Itgax
Fam175b
2700050L05Rik

Zc3h3
Zfp583
Vmn2r29
Vmn2r54
Zfp329
Gltscr1
Dhx34
Apoc1
NA
Catsperg1
Zfp940
Hcst
Gramd1a
C230052I12Rik
Rpl29
Prr12
Saal1
Saa3
Snord116
Klf13
Mef2a
Crtc3
Nmb
2210018M11Rik
Dnajb13
Atg16l2
Socs6
Tmem41b
Eri2
Sh2b1
Taok2
Prrt2
Maz
Rgs10
Ate1
Chst15
R74862
Tnfrsf26
A430078G23Rik
Arhgef18
Zfp358
Tnfsf13b
Fbxo25
Kbtbd11
Tmem66
Rpl29
Cdkn2aip
Aga
Atp6v1b2
Gmip
Lpar2
Mrpl34

Zfp882
Hmgxb4
Podnl1
Lonp2
Zfp821
Hsd17b2
Foxl1
Banp
Tubb3
Clec4g
Rasa3
Erich1
Letm2
Wrn
Psd3
2810422J05Rik
Tmem184c
Usp38
Scoc
Cd97
Ces1b
Acd
Ranbp10
Rpl29
Hp
2310061C15Rik
Cotl1
Fbxo31
Snai3
Irf2bp2
Mmp12
Rpl29
Ubl5
Ets1
BC024479
Snord14d
Thy1
Rdx
Ube2s
Narg2
Anxa2
Gtf2a2
Zfp280d
Mns1
Klhl31
Dopey1
Cep70
Stag1
Rpl29
4921517D21Rik
Elp6
Exog

Slc25a38
5830454E08Rik
Ccr9
Dync2h1
Cdkn2d
Zfp599
Hyls1
1110032A03Rik
Ptpn9
Ppcdc
Pias1
Ptplad1
Fam63b
Tex9
Lrrc1
Snhg5
1190002N15Rik
Atr
Abhd14a
Tdgf1
Ccr4
Slc6a20b
Gm14379
Pim2
Kdm6a
Sh2d1a
Sash3
Bcorl1
Slc25a14
Ppdpf
Xlr4b
Msn
Gpr174
Atg4a
Phf8
Kdm5c
Sh3kbp1
Figf
Mid1
Rpgr
Cfp
Upf3b
Thoc2
Rbmx
Xlr4b
Arhgap4
G6pdx
Atrx
Itm2a
2610002M06Rik
Brwd3
Tspan6

Rgs3
Tceanc
Ssty2
Rmi1
Ccdc82
Nfatc3
