# Supplementary material for: A genome wide transcriptional model of the complex response to pre-TCR signalling during thymocyte differentiation
Source: Oncotarget. 2015 Sep 22;6(30):28646–60. doi: 10.18632/oncotarget.5796 (PMC4745683; doi:10.18632/oncotarget.5796)
Supplement: Supplementary file 6 [file oncotarget-06-28646-s006.pdf]

3110035E14Rik	Gene cluster Late Short
Terf1	
Ly96	
Crispld1	
Paqr8	
Sgol2	
Cflar	
Cyp20a1	
Cd28	
Igfbp2	
Acsl3	
Fbxo36	
Cops7b	
Sh3bp4	
Aspm	
Cenpl	
Vamp4	
Alyref2	
Slamf6	
Kcnj10	
Aim2	
Efcab2	
Ahctf1	
Lin9	
Haus5	
Bpnt1	
Slc30a10	
Nsl1	
Hmgb3	
Cks2	
Tram2	
Prim2	
Bag2	
Sema4c	
Creg2	
1700029F09Rik	
Slc39a10	
Mfsd6	
Pgap1	
Rftn2	
Tmem237	
Mpp4	
Raph1	
Bard1	
Bard1	
A530040E14Rik	
Pde6d	
Hjurp	
Pask	
Pdcd1	
St8sia4	
Pam	

Srgap2
Elf3
Shisa4
Tsen15
Cep350
Dnm3
Scyl3
Nuf2
Ly9
Pea15a
Lbr
Hlx
Cenpf
Fbxo5
Il20ra
Epb4.1l2
Gstm3
9030224M15Rik
Rtkn2
Madcam1
Arid3a
Zbtb7a
Kif14
Dohh
2210404O07Rik
Gm10778
Hcfc2
Ccdc53
4933408J17Rik
Ube2n
Gm5176
Mdm1
Tmem194
Prim1
1700052N19Rik
Mthfd1l
Heca
Enpp1
Cdk1
Phyhipl
Prmt2
Pcnt
Tcf3
Mknk2
Mob3a
Fzr1
Sirt6
Zfp781
Nfyb
Appl2
4930547N16Rik
Gas2l3

Actr6
Nedd1
Plxnc1
4930430F08Rik
Syt1
Glipr1
Kcnmb4
Nup107
Ykt6
Nudcd2
Il5
Hist3h2ba
A030009H04Rik
Gps2
Ybx2
Mis12
Rtn4rl1
5430435G22Rik
Spag5
Ppm1d
Mmd
Rara
Tubg1
Rdm1
BC030867
Mdk
Taco1
Sox9
Birc5
Cbx2
Rac3
Hmga1
Slc16a3
Fignl1
Ahsa2
Chac2
Hmmr
Ublcp1
0610009B22Rik
Atox1
Hist3h2bb-ps
Zkscan17
Cenpv
Atp1b2
2810408A11Rik
Cspp1
Gsg2
Srr
Eral1
Dhrs11
Ppm1d
Brip1

2810422O20Rik
Ints2
Prr11
Rad51c
Acsf2
Eme1
Xylt2
Tbx21
Fbxo47
Top2a
Aarsd1
Vat1
Hdac5
Kif18b
Arhgap27
Limd2
Smurf2
Pitpnc1
Cacng4
Gm11711
Galk1
Wbp2
Tk1
Actg1
Arhgdia
Pycr1
Kif3c
2410018L13Rik
Mboat2
Agr2
Dock4
G2e3
Brms1l
Tmx1
1700009P17Rik
Lrrc9
Syne2
Arg2
Rad51l1
Acot1
Dnalc1
Gpr65
Tdp1
Serpina3n
Evl
Meg3
Zfp386
Ncapg2
Cenpo
Ncoa1
Gen1
Vsnl1

Dld
Zfp277
Nova1
Egln3
Mis18bp1
1110034A24Rik
Sav1
Trim9
Rtn1
Dhrs7
Mlh3
Cspp1
4930534B04Rik
Efcab11
Gpr68
Hmgb1
Amph
Hist1h3g
Hist1h2bm
Hist1h4m
Hist1h2be
Hist1h4h
Hist1h3g
1190005F20Rik
Hist1h4d
Hist1h1t
Hist1h2bb
Hist1h4b
Hist1h1a
Cap2
Cks2
4732471D19Rik
Cbx3
Lpcat1
2210408I21Rik
Lysmd3
Poc5
Gfm2
Depdc1b
Gpank1
Stard3nl
Zkscan3
Hist1h2br
Hist1h4b
Hist1h2ak
Hist1h1b
Hist1h2an
Hist1h2br
Hist1h4n
Hist1h2ao
Zfp322a
Hist1h2bh

Hist1h4f
Hist1h2ae
Hist1h4c
Hfe
Hist1h2ba
Tpmt
Dek
Cenpp
Gprin1
Mxd3
H2afy
Tifab
6720489N17Rik
AA987161
Zfp738
Zfp71-rs1
Trip13
Iqgap2
Hmgcr
Plp2
Hexb
Cenph
Gapt
Map3k1
Hmgcs1
Abhd6
Ptprg
Fdft1
Vcl
D14Abb1e
Glt8d1
Pbrm1
Cdkn3
Cgrrf1
Abhd4
Mrpl52
Lrp10
Dcaf11
Khnyn
Ccni
Cab39l
Trim13
Pbk
1300010F03Rik
Gm5465
Sugt1
6720463M24Rik
B930095G15Rik
Cadps
Gng2
Plac9
Txndc16

Dlgap5
Cideb
Ska3
Extl3
Esco2
Cdca2
Adamdec1
Mir687
Akap11
Diap3
Pabpc4
Tgds
2410089E03Rik
Fam134b
Sema5a
Med30
Wdyhv1
Zfp623
Cks2
Commd5
Csdc2
Fbln1
Gtse1
Pim3
Cntn1
Troap
Espl1
Il7r
Trio
Rad21
Has2
Atad2
Tmem65
Eif2c2
Eif2c2
Zfp647
Ift27
Cbx7
Rangap1
Cenpm
Mapk11
Plxnb2
Cpt1b
Chkb
Kif21a
Yaf2
Rhebl1
Fmnl3
Gpr84
Glis2
Dnaja3
Ntan1

2900011O08Rik
Bex6
Tm4sf19
Tnk2
Lsamp
Cd200r1
Cd200r2
C330027C09Rik
Jam2
Olig2
Il10rb
Coro7
Etv5
Rfc4
Bcl6
Slc35a5
Trat1
Psmc1
5330426P16Rik
Nxpe3
2610039C10Rik
Dscam
Gm6712
Zfp946
Pgp
Mlst8
Pacsin1
Pde9a
Pknox1
Mdc1
Zfp57
Plcl2
Ddx11
Zfp161
Lbh
Heatr5b
Qpct
Galm
Gemin6
Slc22a3
Tpm4
Paqr4
Ccnf
Chtf18
Wdr90
Fam195a
Snrpg
AI413582
Ppil1
D17H6S56E-5
Tcf19
Cenpq

Daam2
Sgol1
Tubb4a
C3
Myl12b
Ndc80
Pigf
Pnkd
Kpna2
Nrxn1
Chmp3
Kif20a
Pura
Stk32a
Lmnb1
Csf1r
Mro
Mbp
Rttn
Cd226
Cdh2
B4galt6
Reep5
C330018D20Rik
Spire1
Ska1
Haus1
Slc14a1
1810055G02Rik
B3gnt1
Cdca5
Men1
Slc22a8
Fam111a
Lpxn
Nmrk1
Apba1
Smarca2
Vldlr
Kif20b
Kif11
Cep55
Tmem20
Tbc1d12
Ina
Taf5
Shoc2
Rad9
Cnih2
Ovol1
Incenp
Tmem138

Slc1a1
Pank1
Ppp1r3c
Arhgap19
4930506M07Rik
Psd4
Odf2
Fam102a
Ak1
Sh2d3c
Psmd14
Klhl23
Nup35
Fam171b
Sfpi1
Ckap5
Kif18a
Ccdc34
Lpcat4
Emc7
BC052040
Bub1b
5430417L22Rik
Casc5
Nusap1
Tmem62
Vps16
Tpx2
Nnat
Ppp1r16b
Ube2c
Rbm38
Nsun6
Arhgap21
4933433C11Rik
Tubb4b
Psmd5
Phf19
Galnt3
Spc25
Nfe2l2
Arhgap11a
Oip5
Frmd5
Gatm
Ncaph
Bub1
Ckap2l
4930402H24Rik
Rassf2
Rbbp9
Ralgapa2

Zfp334
Zfp217
Bcas1
Aurka
Rps29
Lrrcc1
Mfn1
Atp11b
Plk4
3110057O12Rik
3110057O12Rik
Dclk1
Arhgef11
Arhgef2
Pmvk
Crtc2
Otud7b
Hist2h2ab
Sort1
Slc25a24
Extl2
Elovl6
Ppa2
Cenpe
Usp33
Depdc1a
Pex2
Hmgb1
Cpa3
Kpna2
Ect2
Ccna2
Exosc8
Arfip1
Bcan
Pmf1
Efna3
Prune
Anxa9
Hist2h2aa1
Hist2h3c2
Ttf2
Ptgfrn
Cd53
Gstm1
Gpsm2
Fam102b
Amy2a5
Cdc14a
Ank2
Clvs1
1110037F02Rik

Rad54b
Rbm12b2
Rragd
Serf2
Melk
Grhpr
Zfp189
Smc2
Ift74
Raver2
Stil
Fhl3
Clspn
Fabp3
Paqr7
2610002D18Rik
Hmgb2
Eno1
A430005L14Rik
Pex10
Cpsf3l
9430015G10Rik
Ints8
Atp6v0d2
2610029I01Rik
Manea
3110043O21Rik
Exosc3
Abca1
Ptpn3
Ptpn3
Ptpn3
Ptpn3
Ptpn3
Ptpn3
Ptpn3
Ptpn3
Ptpn3
Wdr31
Elavl2
Itgb3bp
Cpt2
Cc2d1b
Plk3
Kif2c
Szt2
Cdc20
Ccnb1
Dem1
Cdca8
Iqcc
Phactr4

Med18
Stx12
Trnp1
Cd52
Tceb3
Hmgb2
Hmgb2
Rpl29
2610305D13Rik
Chchd2
Kif1b
Nmnat1
Camta1
Trp73
Gltpd1
Tubb4b
4930420K17Rik
Gm8944
Magi2
Insig1
Mapre3
Tacc3
Jakmip1
Crmp1
Ncapg
Uchl1
Slc30a9
Fgf5
Cdc7
Chek2
Ttc28
Coq5
Cit
P2rx4
Kntc1
Aacs
Dtx2
Trrap
Gsx1
Brca2
Dbf4
Reln
Atp5l
Tmem129
Haus3
Nsg1
Zfp518b
Slain2
Scarb2
Lin54
Wdfy3
Mapk10

Barhl2
Fam69a
Vmn2r14
Mlec
Cox6a1
Tpcn1
Oas1c
Rhof
Cdk2ap1
Psph
Taf6
BC037034
Prkar1b
Mtif3
Zfp141
Gimap9
Gimap7
Gpnmb
D330028D13Rik
Mpp6
Snx10
Chn2
Plekha8
Mad2l1
Foxi3
Krcc1
Reep1
Mgll
Chl1
Fancd2
Syn2
Raf1
Cand2
Gemin6
Rasgef1a
Tuba8
Foxj2
Clec4a3
Cdca3
Foxm1
Clec2i
Clec2d
Clec12a
Bet1
Sgce
Rpa3
Pot1a
Chchd3
Hipk2
Jhdm1d
Clec5a
4921507P07Rik

1700019G17Rik
Aplf
Erc1
Erc1
Eno2
Cd9
Hist4h4
Tsen34
Zfp524
Zscan4c
Zfp110
Zfp128
Fbxo46
Tmsb10
Zfp626
Sirt2
Zfp790
Zfp420
Cox7a1
Hspb6
Luzp2
Fanci
5730590G19Rik
Pde8a
Mex3b
Pgm2l1
Rrm1
Zfp143
Spon1
6330503K22Rik
Plk1
Il21r
Mapk3
Itgam
Ppp2r2d
Syt5
Ube2s
Zfp954
Rnf141
Atp1a3
Mrps12
Tbcb
Rps12
BC053749
Lin37
Ccne1
Zfp473
Bax
Dhdh
E2f8
Depdc1a
Snord116

Blm
Tmem126b
Mrps36
Rnf141
Nup35
Igsf6
Nsmce1
Nfatc2ip
Aldoa
2900092E17Rik
Kif22
Zfp747
Stx1b
Cox6a2
Mki67
Ebf3
Lrdd
Ascl2
Tnfrsf23
Fadd
Kbtbd11
Purg
Gtf2e2
Zdhhc2
Enpp6
Scrg1
Hmgb2
Lpl
D130040H23Rik
Rab3a
Tpm4
Asf1b
Prkaca
Gadd45gip1
Gpt2
Gnao1
Mt2
Mt1
Ap3s1
Gins3
Fbxl8
Slc9a5
St3gal2
Cenpn
Trim67
Angpt2
Xkr5
Ckap2
Brf2
Rbpms
Eri1
Ifi30

Mast3
Hmgn2
Il27ra
Rpgrip1l
Tradd
Fhod1
Pdxp
Tmem231
Fanca
Ncapd3
Hepacam
Siae
H2afx
Slc37a4
Zw10
Pih1d2
Arhgap20
Pif1
Rfx7
Myo5a
Ttk
Nt5e
Mthfs
Tmem41b
Twf2
Poc1a
Hyal1
Mtap4
Cspg5
Stt3b
Kif15
Sacm1l
Zfp560
Zfp426
Spc24
Anln
Fli1
AW551984
C2cd2l
Il10ra
Dmxl2
Snx33
Scamp5
Senp8
Lrrc49
Coro2b
Zwilch
Aph1b
Tpm1
Ccnb2
Fam63b
Mras

Rad54l2
Slc38a3
Gm2799
Praf2
Zcchc12
Rab33a
Tktl1
Plxna3
F630028O10Rik
Kif4
Uprt
Tlr13
Cenpi
Gprasp2
Bex4
Smc1a
Fancb
Aifm1
Fam122b
BC023829
Flna
Gyk
Pola1
Pja1
Pdzd11
Ercc6l
1700031F05Rik
Hmgn5
Dcx
Prdx4
Rab9
Vamp7
Sly
Ublcp1
A730008H23Rik
Zfp429
Cd99
